# Ibuprofen versus pivmecillinam for uncomplicated urinary tract infection in women—A double-blind, randomized non-inferiority trial

**DOI:** 10.1371/journal.pmed.1002569

**Published:** 2018-05-15

**Authors:** Ingvild Vik, Marianne Bollestad, Nils Grude, Anders Bærheim, Eivind Damsgaard, Thomas Neumark, Lars Bjerrum, Gloria Cordoba, Inge Christoffer Olsen, Morten Lindbæk

**Affiliations:** 1 Department of Emergency General Practice, Oslo Accident and Emergency Outpatient Clinic, Oslo, Norway; 2 Antibiotic Centre of Primary Care, Department of General Practice, Institute of Health and Society, University of Oslo, Oslo, Norway; 3 Division of Medicine, Stavanger University Hospital, Stavanger, Norway; 4 Department of Medical Microbiology, Vestfold Hospital Trust, Tønsberg, Norway; 5 Department of Global Public Health and Primary Care, University of Bergen, Bergen, Norway; 6 Bergen Accident and Emergency Department, Bergen City Council, Bergen, Norway; 7 Primary Health Care and Planning Division, Kalmar County Council, Kalmar, Sweden; 8 Section of General Practice and Research Unit of General Practice, Department of Public Health, University of Copenhagen, Copenhagen, Denmark; 9 Research Support Services CTU, Oslo University Hospital, Oslo, Norway; Stanford University, UNITED STATES

## Abstract

**Background:**

Although uncomplicated urinary tract infections (UTIs) are often self-limiting, most patients will be prescribed antibiotic treatment. We assessed whether treatment with ibuprofen was non-inferior to pivmecillinam in achieving symptomatic resolution by day 4, with a non-inferiority margin of 10%.

**Methods and findings:**

This was a randomized, controlled, double-blind non-inferiority trial. We recruited patients from 16 sites in a general practice setting in Norway, Sweden, and Denmark. Non-pregnant women aged 18–60 years presenting with symptoms of uncomplicated UTI were screened for eligibility from 11 April 2013 to 22 April 2016. Patients with informed consent were randomized (1:1 ratio) to treatment with either 600 mg ibuprofen or 200 mg pivmecillinam 3 times a day for 3 days. The patient, treating physician, and study personnel were blinded to treatment allocation. The primary outcome was the proportion of patients who felt cured by day 4, as assessed from a patient diary. Secondary outcomes included the proportion of patients in need of secondary treatment with antibiotics and cases of pyelonephritis. A total of 383 women were randomly assigned to treatment with either ibuprofen (*n =* 194, 181 analyzed) or pivmecillinam (*n =* 189, 178 analyzed). By day 4, 38.7% of the patients in the ibuprofen group felt cured versus 73.6% in the pivmecillinam group. The adjusted risk difference with 90% confidence interval was 35% (27% to 43%) in favor of pivmecillinam, which crossed the prespecified non-inferiority margin. Secondary endpoints were generally in favor of pivmecillinam. After 4 weeks’ follow-up, 53% of patients in the ibuprofen group recovered without antibiotic treatment. Seven cases of pyelonephritis occurred, all in the ibuprofen group, giving a number needed to harm of 26 (95% CI 13 to 103). Five of these patients were hospitalized and classified as having serious adverse events; 2 recovered as outpatients. A limitation of the study was the extensive list of exclusion criteria, eliminating almost half of the patients screened. We did not register symptoms in the screening process; hence, we do not know the symptom burden for those who declined to participate. This might make our results less generalizable.

**Conclusions:**

Ibuprofen was inferior to pivmecillinam for treating uncomplicated UTIs. More than half of the women in the ibuprofen group recovered without antibiotics. However, pyelonephritis occurred in 7 out of 181 women using ibuprofen. Until we can identify those women who will develop complications, we cannot recommend ibuprofen alone as initial treatment to women with uncomplicated UTIs.

**Trial registration:**

ClinicalTrials.gov NCT01849926

EU Clinical Trials Register (EU-CTR), EudraCT Number 2012-002776-14

## Abstract 2

In a multicenter trial, Ingvild Vik and colleagues studied a non-antimicrobial treatment for urinary tract infections in women.

## Author summary

**Why was this study done?:**

- Most uncomplicated urinary tract infections (UTIs) are self-limiting, but they are commonly treated with antibiotics as this gives quicker symptom relief.
- In 2010 a German study showed that two-thirds of women who received initial treatment with ibuprofen recovered without taking antibiotics.
- Antibiotic resistance is a serious public health problem, mainly caused by overuse of antibiotics. We performed a trial to explore whether treatment with ibuprofen could be a safe and efficient alternative to antibiotics in women with uncomplicated UTIs.

**What did the researchers do and find?:**

- Between April 2013 and June 2016 we recruited 383 women with symptoms of uncomplicated cystitis, who were randomized to receive a 3-day treatment of either ibuprofen or pivmecillinam (an antibiotic commonly used to treat UTIs in Scandinavia).
- It took 3 days longer for the patients in the ibuprofen group to get well.
- In the ibuprofen group, 46% of the patients returned to the doctor within 4 weeks with persisting, worsening, or new symptoms; 10% of the patients in the pivmecillinam group came back for a second consultation.
- Twelve patients (6.6%) developed a febrile UTI; all were initially treated with ibuprofen. In 7 of these patients (3.9%), the infection spread to the kidneys (pyelonephritis). All patients recovered with antibiotic treatment.
- More than half of the patients initially treated with ibuprofen got well without taking antibiotics. Looking at only the patients who got well without needing a subsequent prescription for antibiotics, the women in the ibuprofen group had symptoms for a median of 4 days, 1 day longer than the patients initially treated with antibiotics.

**What do these findings mean?:**

- Antibiotic treatment is the best treatment we have for uncomplicated UTI.
- Until we can identify the patients who need antibiotic treatment to avoid complications, we cannot recommend ibuprofen alone to women with uncomplicated UTI.

## Introduction

More than half of all women will experience an uncomplicated urinary tract infection (UTI) during life [1], and 20% will have recurring infections [2]. The condition is mostly self-limiting and rarely progresses to an upper UTI [3,4]. Despite this, most women who see a doctor are prescribed antibiotic treatment, as it is known to give quick symptom relief and shorten the course of the condition. Some general practitioners (GPs) in Scandinavia use a urinary dipstick result to confirm a UTI, but if the clinical suspicion of the diagnosis is there, a negative dipstick result is not considered a good method to rule out infection. In Norway the guidelines state that the diagnosis of uncomplicated UTI can be given based on symptoms alone [5]. A Norwegian study showed that prescribing for uncomplicated UTI that was delegated to a nurse who followed a diagnostic algorithm provided the same outcome as a doctor’s consultation [6].

UTIs are the second most common cause for antibiotic prescribing in general practice [7]. With antibiotic resistance on the rise, it is crucial to reduce unnecessary antibiotic use [8,9]. Antibiotics can also cause unpleasant and potentially severe side effects, and so avoiding unnecessary use would be beneficial to patients [10,11]. A meta-analysis of placebo-controlled randomized trials of uncomplicated UTI showed that antibiotics were superior to placebo in achieving both symptomatic and bacteriological cure [12]. Overall, there was no significant difference in development of pyelonephritis.

A small randomized pilot study from Germany was published in 2010 by Bleidorn et al. [13]. Their results suggested that ibuprofen was non-inferior to ciprofloxacin for achieving symptomatic cure in uncomplicated UTI. They went on to conduct a larger trial to confirm their findings, evaluating whether the use of antibiotics (fosfomycin) for uncomplicated UTI could be reduced by giving initial treatment with ibuprofen. This inspired a Swiss group of researchers to start a trial comparing diclofenac to norfloxacin in the treatment of uncomplicated UTI, and we started planning a Scandinavian trial. The larger German trial, by Gagyor et al., was published in December 2015 [14]. The Swiss trial, by Kronenberg et al., was published in November 2017 [15]. Both trials showed that antibiotic treatment was superior to treatment with nonsteroidal anti-inflammatory drugs (NSAIDs).

In this paper we report the results from our trial comparing ibuprofen to pivmecillinam for uncomplicated UTI. The main objective of this study was to assess whether ibuprofen was non-inferior to antibiotics (pivmecillinam) in achieving symptomatic cure by day 4 in uncomplicated UTI. A 10% non-inferiority margin was established based on clinical expertise. We also wanted to assess the occurrence of complications.

## Methods

### Trial design and participants

The study was a double-blind, randomized, parallel group, multicenter non-inferiority trial. Patients were randomized in a 1:1 ratio to treatment with either ibuprofen or pivmecillinam.

All patients received oral and written information about the trial and signed an informed consent form. The trial was conducted in compliance with the International Conference on Harmonisation guidelines for good clinical practice (GCP) and the Declaration of Helsinki. The trial was approved by the Regional Ethical Committee in Norway (REK) and the Norwegian Medicines Agency (SLV) (2012/1569 C), the Committee on Biomedical Research Ethics for the Capital Region of Denmark (H-4-2013-145), the Regional Ethical Committee in Lund, Sweden (Dnr 2014/28), and the Medical Products Agency, Uppsala, Sweden (Dnr 5.1-2014-12072). Safety data were monitored throughout the trial by a certified monitor in each country. There was no independent data monitoring committee for the trial. All study personnel were trained in GCP. The study was conducted and analyzed according to the protocol (S1 Protocol) and the statistical analysis plan (SAP) (S1 Appendix).

We recruited non-pregnant women aged 18–60 years with symptoms of an uncomplicated UTI. Inclusion criteria were dysuria combined with either increased urinary frequency or urinary urgency or both, with or without visible hematuria. The exclusion criteria were duration of symptoms for more than 7 days; allergies/adverse reactions to penicillin or ibuprofen; breastfeeding a child under 1 month of age; any sign of an upper UTI (fever, upper back pain, reduced general condition); vaginal irritation/discharge; severe abdominal pain; diabetes; kidney disease; genetic aciduria; severe gastritis; ulcerative colitis; Crohn's disease; low platelets; use of probenecid, steroids, immunosuppressant drugs, or blood-thinning drugs; previous pyelonephritis; use of urinary catheter or symptoms of a UTI within the last 4 weeks; or use of antibiotics within the last 2 weeks (S1 Fig). Ability to give written consent was assessed by the study nurse or study doctor. Potential participants were excluded if they were assessed as having severe psychiatric illness, dementia, severe drug addiction, or inability to communicate in the official language of the country.

Patients were recruited from the accident and emergency outpatient clinics (AEOCs) in Oslo and Bergen, Norway, 7 general practices in Denmark, and 7 general practices in Sweden. The AEOCs in Norway are part of the primary healthcare system and are mainly staffed with GPs or doctors training to become GPs; we therefore consider them to be a general practice setting. Women presenting with symptoms of an uncomplicated UTI were consecutively screened for eligibility by a doctor or a study nurse using a questionnaire (S1 Fig). The questionnaire consisted of the inclusion and exclusion criteria and was based on the diagnostic algorithm already in use at the AEOC in Oslo [6].

A pilot study was performed at each site to test logistics: 30 patients were enrolled at the AEOC in Oslo, 10 patients at each of the other sites. We did not make any major changes to the protocol after these pilots; only 2 exclusion criteria were added (Crohn's disease and ulcerative colitis). The exclusion criteria were added when a patient with Crohn's disease wanted to be enrolled in the study. We had not thought of this patient group when writing the protocol, but when the issue emerged, we decided to exclude patents with inflammatory bowel disease. The patients from the pilot studies were included in the main study.

### Blinding and randomization

A Norwegian pharmaceutical preparation company, Kragerø Tablettproduksjon, over-encapsulated the medicine used in the study. They used gelatin capsules where red iron oxide was used for color and titanium oxide as an opacifier. The final product was tested before the trial started to ensure that the capsules had the same look, feel, weight, and taste. The study medicine was packed in 2 different kits, one with 9 capsules containing 200 mg pivmecillinam each, the other with 9 capsules containing 600 mg ibuprofen each. Each kit was labeled with a study number. The labeling was done following a computer-generated randomization list created by an independent statistician using randomized block sizes of 2, 4, 6, or 8, stratified by country [16]. Different study numbers were assigned to each study site: Oslo, 1000–1299; Bergen, 1300–1399; Denmark, 1400–1499; and Sweden, 1500–1599. The list linking the study number to the active substance was kept at Kragerø Tablettproduksjon. The list was retrieved only at the end of the study, when all data had been collected and entered into the study database, and the SAP was completed and signed.

Following the randomization list, Kragerø Tablettproduksjon distributed the study medicine to each study site, where it was kept in a locked facility. At inclusion, each patient received a kit labeled only with the study number. Neither the research nurse/doctor nor the patient knew which active substance was given, thus resulting in blinding for both the nurse/doctor and patient.

### Interventions/procedures

The patients were consecutively allocated to active treatment with either pivmecillinam 200 mg or ibuprofen 600 mg 3 times a day for 3 days. They received a kit labeled with a study number; the kits were retrieved in chronological order from a locked facility at each study site. They were all given routine advice to stay hydrated alongside information on proper self-care. They received a patient diary for recording daily symptoms and on which day they felt completely cured. The diary was based on a previously validated version [17,18]. The patients were asked to record possible adverse effects or complications in the diary. They were also asked to record whether they had completed the treatment, and, if they had not, they were asked how many capsules they had left.

Except for the drugs listed in the exclusion criteria, there are no known significant drug interactions for the study medications. The patients were directed to continue taking all prescription drugs as usual throughout the trial and to record all prescription and non-prescription drugs in the patient diary. They were not allowed to take any additional NSAIDs during the trial. They were allowed to take paracetamol if they needed additional pain relief. When the patients had completed the diary, they were asked to return it to the study site.

A research nurse/doctor contacted the patients for a telephone follow-up after 14 and 28 days. In the first follow-up, the patients were asked if they felt cured, and if so, how many days it had taken; they were asked this question again in the second follow-up if they answered “no” in the first follow-up. In both follow-ups, they were asked if they had experienced a relapse of symptoms, if they had seen a doctor again, and if they had received antibiotic treatment.

The monitors visited the study sites at the start of the pilot and after the pilot, and made 1 or 2 visits during the study and a final visit when all patient follow-up was done.

### Microbiology/laboratory analyses

The results of a urine dipstick (leukocytes, protein, nitrite, and blood) were recorded at inclusion. The dipstick results were not part of the inclusion criteria, but were recorded to provide baseline information for later predictor analysis. A urine sample was sent to the local microbiology laboratory for culturing. In Norway and Denmark, the urine sample was transported in a plastic container with boric acid. In Sweden, study personnel used plastic containers without any added chemicals; this was due to short delivery time to the laboratory (<6 hours).

The patients received equipment to take a second urine sample at home and send it to the laboratory for a new culture after 2 weeks. They were instructed to collect a midstream urine sample after spreading of the labia, preferably morning urine. They could send the urine sample by mail in a freepost envelope or deliver it to the study site.

The uropathogens were quantified in colony-forming units per milliliter. Significant bacteriuria was defined according to current European guidelines for patients with symptoms of UTI as ≥10^3^ /ml for primary pathogens, ≥10^4^/ml for secondary pathogens, and ≥10^5^/ml for doubtful pathogens [19]. Clinical breakpoints for susceptibility were assessed according to the European Committee on Antimicrobial Susceptibility Testing (EUCAST) [20].

### Second medical consultation

If a patient experienced getting worse or not getting better, she was instructed to contact either the study doctor for advice or the study site for a second medical consultation. Contact information and routines were described in the consent form. There were written routines at each study site for health personnel to follow when a study patient came back for a second medical consultation.

Patients who received additional treatment, had an allergic reaction, or were admitted to the hospital remained in the study unless they expressed a wish to withdraw their consent. Their data were included in the analyses according to the principle of intention to treat.

### Safety/unblinding

In case of immediate need for unblinding, sealed, opaque envelopes for each study number were kept at each study site. Each envelope contained the name of the active substance given to the patient with the corresponding study number. If necessary, the envelope would be opened, and the patient and the doctor treating the patient would be informed which active substance the patient had received.

If more than 10 patients within the first 100 patients included were hospitalized, this would be a cause for unblinding of the hospitalized patients. If it turned out that significantly more patients in the ibuprofen group were hospitalized, we would consider terminating the study.

The trial by Gagyor et al. was published in December 2015 [14]. We realized that the results of their main trial were in favor of antibiotic treatment, but with 67% in the ibuprofen group recovering with only 1 more day of symptoms, we were still optimistic regarding the outcome of our trial. We did notice that Gagyor et al. had more cases of pyelonephritis in the ibuprofen group, but because the rate was only 2.1% and the study was not powered to detect significant differences for pyelonephritis, we did not consider this to be a reason for early termination of our trial. The trial by Kronenberg et al. was published after all our analyses were finalized and did not have any impact on the conduct of our trial [15].

### Outcomes

All outcomes are similar to outcomes chosen in previous studies demonstrating efficacy of antibiotic treatment for uncomplicated UTI [12,14,21].

The main outcome measure of this trial was the proportion of patients who felt cured by day 4 as recorded in the patient diary. If we did not have information from the diary, we used the number of days until cure reported by the patient during the telephone follow-up.

Secondary outcome measures included duration of symptoms and the patients’ symptom load for specific symptoms. We calculated a daily symptom sum score for each patient from the patient diary. The sum score consisted of the symptoms dysuria, urinary urgency, and urinary frequency. Each symptom was graded on a scale from 0 to 6 where 0 was “normal/not affected” and 6 was “as bad as it could be”, so the maximum potential sum score each day was 18.

Further secondary outcomes were the proportion of patients (i) with positive second urine culture (significant bacteriuria), (ii) in need of a medical consultation within the 4 weeks of follow-up, and (iii) who received antibiotic treatment during this period. Regarding safety, we were specifically interested in how many patients developed an upper UTI (pyelonephritis/febrile UTI) and how many patients experienced adverse events and serious adverse events (SAEs) (defined as any event leading to hospitalization). Pyelonephritis was defined as symptoms of UTI with flank pain, self-reported fever, and C-reactive protein (CRP) ≥ 40 mg/l. If the patient had flank pain and self-reported fever but CRP < 40 mg/l, she was characterized as having a febrile UTI. Pyelonephritis is sometimes also classified as a febrile UTI [22], but in this study we use the term febrile UTI to classify a UTI with less severe systemic impact than pyelonephritis. In the trial by Gagyor et al., they left this classification to the discretion of the GP. In our study, the distinction between febrile UTI and pyelonephritis was not described in the protocol. The doctors were not aware of the 2 different classifications during the study, and we had to rely on their clinical notes in the patient record and the CRP value to make the classifications. We had not predefined flank pain; hence, we decided that this would include both where the doctor perceived the patient as having flank pain upon examination and where patients self-reported flank pain. The classification was done after the trial by Gagyor et al. was published (December 2015) and was clearly defined in the SAP and implemented in the database prior to allocation unblinding.

The main outcome was tested according to non-inferiority, the secondary outcomes according to superiority.

### Sample size and statistical analyses

Assuming no difference between the treatment groups in the proportion of patients feeling cured after 4 days, we calculated that 316 patients (158 in each arm) were required in the primary full analysis set (FAS) analyses to be 80% confident that the 1-sided 95% confidence limit would exclude a difference in favor of pivmecillinam of more than 10%. The anticipated rate of feeling cured after 4 days was 85% in both groups. Assuming a dropout rate of approximately 20%, we aimed to randomize 400 patients.

The null hypothesis of this study was that treatment with ibuprofen would be inferior to pivmecillinam regarding the proportion of patients feeling cured after 4 days by a 10% margin. The alternative hypothesis was that ibuprofen would be non-inferior regarding the proportion of patients feeling cured after 4 days by at most 10%.

The primary efficacy analyses were performed in the FAS, consisting of all randomized patients with at least 1 efficacy assessment after randomization. Secondary efficacy analyses were performed in the per protocol set, consisting of patients in the FAS with a treatment compliance rate of at least 80%.

The primary and secondary dichotomous endpoints were analyzed using logistic regression (single measurement) or mixed effects logistic regression (repeated measurements) with treatment as a fixed effect, adjusted for randomization stratum (center in Norway, country otherwise). Efficacy measures were adjusted risk difference and adjusted relative risk using the delta method.

Continuous endpoints were analyzed using linear mixed models with patient-specific random intercept and treatment, time (randomization to day of assessment), treatment–time interaction, baseline value, and randomization stratum as fixed factors. Time to event endpoints were analyzed using a Cox regression model adjusted for randomization stratum and are presented using Kaplan–Meier plots.

The polytomous complications variable was analyzed using Fisher’s exact methods for contingency tables and Newcombe’s hybrid score method for calculation of risk difference confidence intervals. These analyses were decided upon after unblinding of the data, which revealed the low cell count in the pivmecillinam group. Thus, these analyses should be regarded as exploratory.

Subgroup analyses compared the patients with positive baseline urine cultures to those with negative cultures within the 2 treatment groups, both with regards to symptom load and duration of symptoms. Within the patients with positive urine cultures, we also compared those with cultures susceptible to pivmecillinam to those with cultures resistant to pivmecillinam with regards to symptom burden.

The null hypothesis was tested on the 1-sided 5% significance level as predefined in the SAP. Consequently, the primary efficacy estimate is reported with a 90% confidence interval. Efficacy estimates of secondary endpoints are presented with 95% confidence intervals.

There were no missing data for the primary endpoint in the FAS. Missing dichotomous endpoints were imputed with the worse outcome, except for febrile UTI, pyelonephritis, and SAEs. If we had no information on these endpoints, they were interpreted as not present (best case imputation). We did not impute missing outcomes for continuous and time to event endpoints. Missing data for these endpoints were handled by the linear mixed model and the Cox regression model, assuming missing at random and non-informative censoring at the last point of contact. Further statistical details are described in the SAP (S1 Appendix). Statistical analyses were performed using Stata version 14.1.

## Results

### Randomization and baseline characteristics

**Fig 1:**
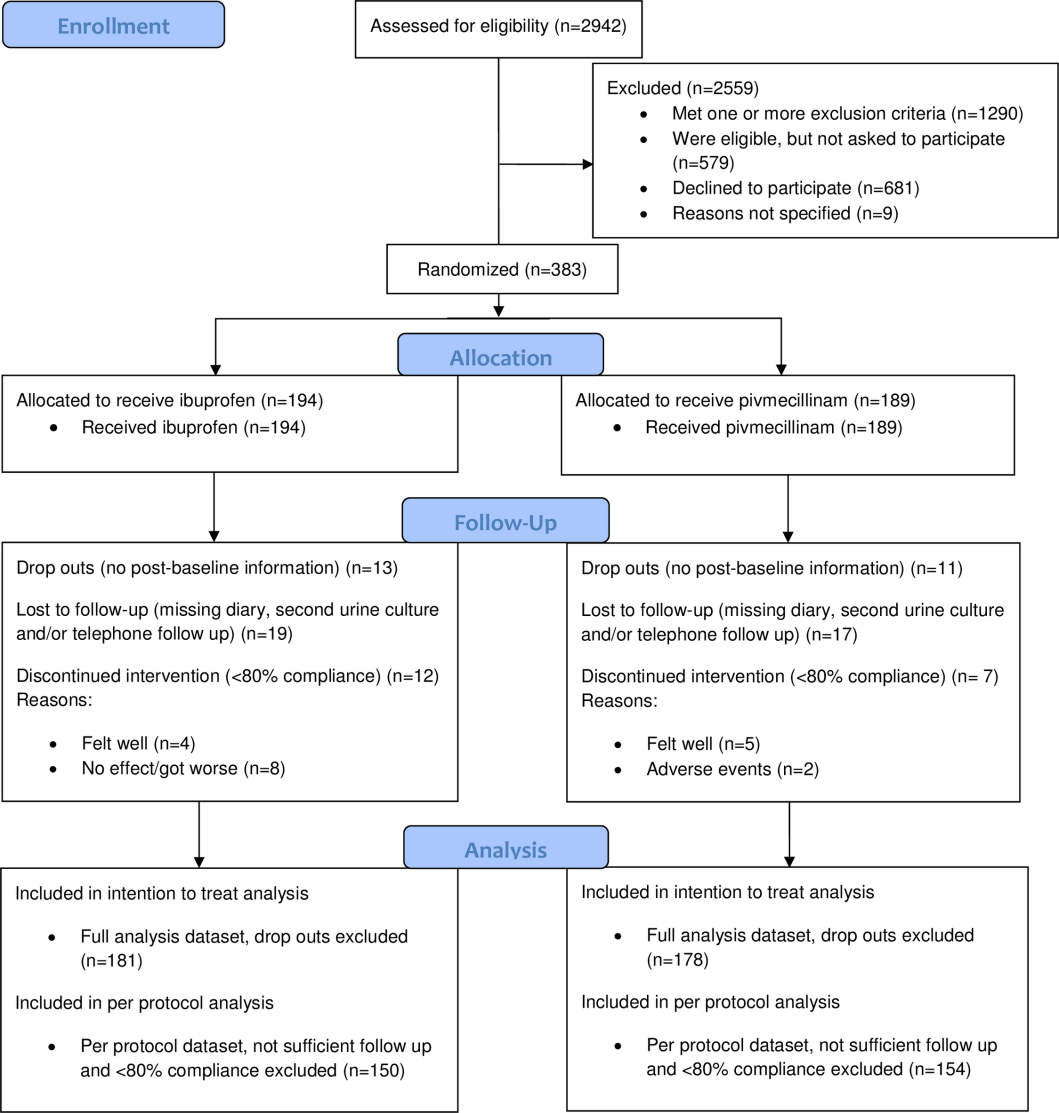
Flow of participants through trial of ibuprofen versus pivmecillinam for women with uncomplicated urinary tract infection.

Patients were assessed for eligibility from 11 April 2013 to 22 April 2016, and the last follow-up was made on 7 June 2016. A total of 2,942 women were screened: 1,290 patients met 1 or more exclusion criteria, 1,269 patients were eligible, and 383 patients were enrolled in the trial (Fig 1). The largest recruitment site was the AEOC in Oslo (260 patients), followed by the AEOC in Bergen (40 patients). In Denmark study personnel recruited 47 patients from 7 sites, and in Sweden they recruited 37 patients from 7 sites. For 24 patients (13 patients in the ibuprofen group and 11 in the pivmecillinam group), we were not able to obtain any post-baseline information, and they were removed from the FAS. The remaining 359 patients were included in the FAS, 181 in the ibuprofen group and 178 in the pivmecillinam group. There were no major differences in baseline characteristics between the 2 groups (Table 1).

**Table 1:** Baseline characteristics for women with uncomplicated UTI randomized to ibuprofen or pivmecillinam.

Characteristic	Ibuprofen (*n =* 181)	Pivmecillinam (*n =* 178)
**Age at enrollment (years), mean (SD)**	28.1 (8.6)	28.5 (10.2)
**Symptom duration at enrollment, *n* (%)**		
<1 day	10 (5.5)	9 (5.1)
1 to 2 days	89 (49.2)	84 (47.2)
>2 to 7 days	80 (44.2)	84 (47.2)
>7 days	2 (1.1)	1 (0.6)
**Signs and symptoms**		
Dysuria, *n* (%)	152 (95.6)	152 (95.6)
Urinary urgency, *n* (%)	157 (98.7)	154 (96.9)
Urinary frequency, *n* (%)	157 (98.7)	156 (98.1)
Visible hematuria, *n* (%)	71 (44.4)	63 (39.6)
Symptom severity sum score*, mean (SD)	12.56 (3.43)	12.29 (3.65)
Dysuria symptom score, mean (SD)	3.92 (1.49)	4.00 (1.48)
Urinary urgency symptom score, mean (SD)	4.42 (1.32)	4.30 (1.46)
Urinary frequency symptom score, mean (SD)	4.21 (1.36)	3.99 (1.41)
**UTIs in last 12 months, *n* (%)**		
0–2 UTIs	141 (90.4)	146 (93.0)
≥3 UTIs	15 (9.6)	11 (7.0)
**Urinary dipstick result positive, *n* (%)**		
Leukocytes	161 (92.5)	165 (93.2)
Blood	135 (77.1)	145 (82.4)
Protein	85 (48.3)	75 (42.6)
Nitrite	35 (19.9)	25 (14.2)
**Urine culture result, *n* (%)**		
Negative/no significant growth	59 (32.8)	64 (36.2)
Positive	121 (67.2)	113 (63.8)
*Escherichia coli*	95 (78.5)	93 (82.3)
*Staphylococcus saprophyticus*	17 (14.0)	12 (10.6)
*Enterococcus faecalis*	2 (1.7)	0 (0.0)
*Klebsiella pneumoniae*	2 (1.7)	2 (1.8)
*Proteus mirabilis*	1 (0.8)	0 (0.0)
*Enterobacter* species	2 (1.7)	4 (3.5)
*Citrobacter koseri*	2 (1.7)	0 (0.0)
Other uropathogens	0 (0.0)	2 (1.8)
Susceptible to pivmecillinam (all pathogens)	93 (76.9)	83 (73.5)
Susceptible to pivmecillinam (*E*. *coli*)	90 (94.7)	81 (87.1)

*Sum of day 0 symptom scores of dysuria, urinary urgency, and urinary frequency, range 0–18. The symptoms were given a value on a scale from 0 to 6, where 0 was “normal/not affected” and 6 was “as bad as it could be”.

SD, standard deviation; UTI, urinary tract infection.

### Outcomes and estimation

**Fig 2:**
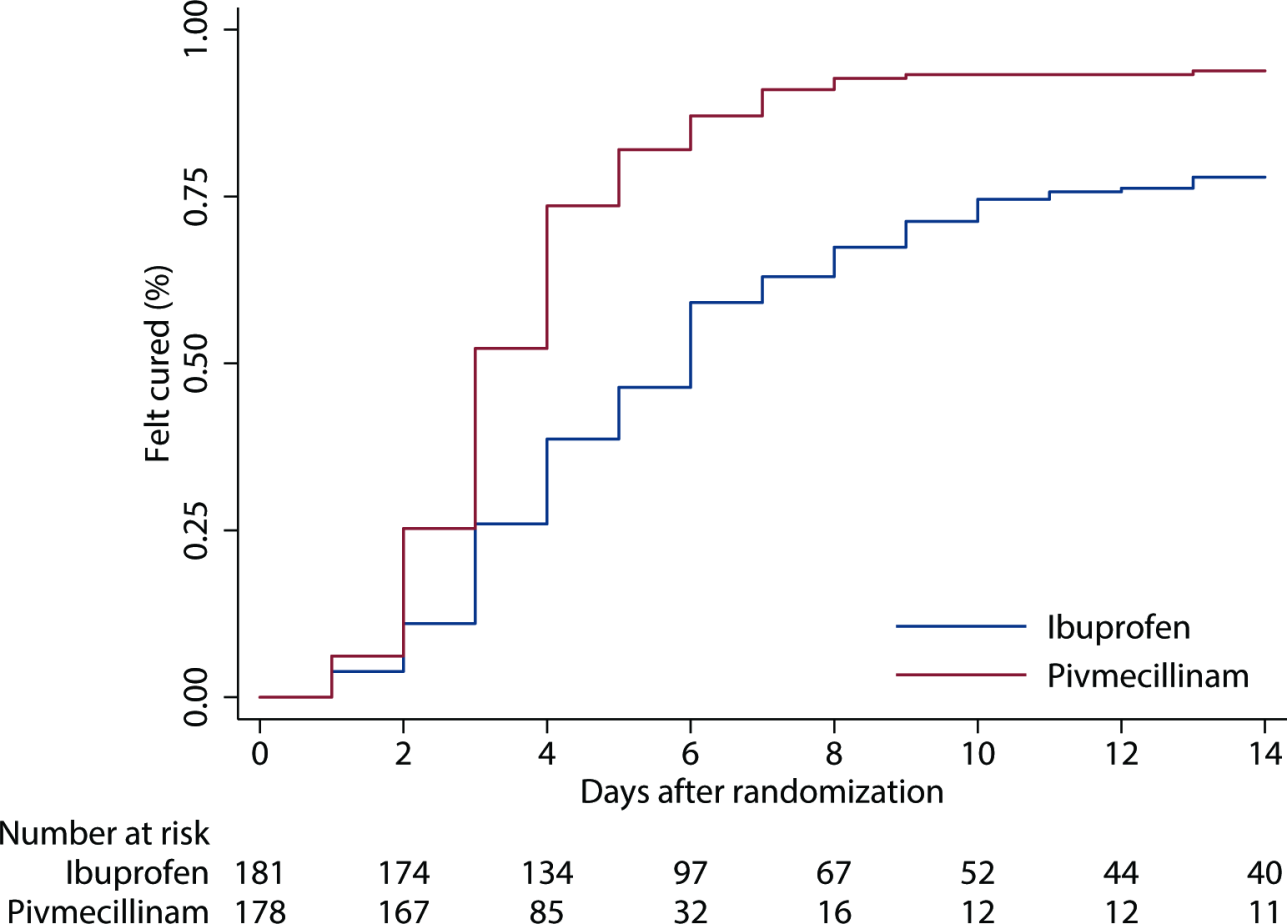
Kaplan–Meyer plot showing the percentage of patients who felt cured on day 0 to 14 by treatment group.

In the ibuprofen group, 70 patients (38.7%) felt cured by day 4 (the primary outcome) versus 131 patients (73.6%) in the pivmecillinam group (Table 2). Adjusted risk difference with 90% CI was 35% (27% to 43%), which is outside the predefined non-inferiority margin, with a non-inferiority test *p*-value of >0.99. The corresponding number needed to treat was 3 (90% CI 2 to 4). Adjusted relative risk was 1.9 (90% CI 1.6 to 2.2). For secondary outcomes the patients in the pivmecillinam group generally felt cured sooner than the patients in the ibuprofen group (Fig 2). Where we did not have data for when the patient felt cured, we assumed that she did not get well within 2 weeks (worst case imputation). The median duration of symptoms was 6 days in the ibuprofen group and 3 days in the pivmecillinam group.

**Table 2:** Summary of primary and key secondary outcomes in women with uncomplicated UTI randomized to either ibuprofen or pivmecillinam: Intention to treat population.

Outcome	Ibuprofen (*n =* 181)	Pivmecillinam (*n =* 178)	Adjusted risk difference (95% CI)
**Primary outcome**			
Patients without symptoms by day 4	70 (39)	131 (74)	35% (27% to 43%)*
**Secondary outcomes**			
Patients without symptoms by day 7	114 (63)	162 (91)	28% (20% to 36%)
Patients without symptoms by day 14	141 (78)	167 (94)	16% (9% to 23%)
Median symptom duration after randomization (days)	6	3	
Urine cultures after 14 days**			
Urine culture positive	43 (28)	16 (10)	−16% (−26% to −7%)
Growth of primary pathogens	29 (19)	6 (4)	−14% (−23% to −6%)
Relapses/complications**			
Secondary treatment with antibiotics by day 14	73 (41)	14 (8)	−32% (−40% to −24%)
Secondary treatment with antibiotics by day 28	83 (46)	18 (10)	−36% (−44% to −27%)
Patients with febrile UTI^†^	5 (3)	0 (0)	−3% (−6% to 0.1%)
Patients with pyelonephritis^†^	7 (4)	0 (0)	−4% (−8% to −1%)
Serious adverse events	6 (3)	1 (1)	−3% (−6% to 0.1%)

Figures are number of women (percentage) unless stated otherwise.

*The primary outcome is presented with 90% confidence interval as per protocol and statistical analysis plan.

**Numbers represent observed (non-missing) data while estimates are based on imputed data.

^†^Complications categorized as no complications, febrile UTI, and pyelonephritis. Treatment differences are presented with unadjusted confidence limits using Newcombe’s hybrid score method.

UTI, urinary tract infection.

**Fig 3:**
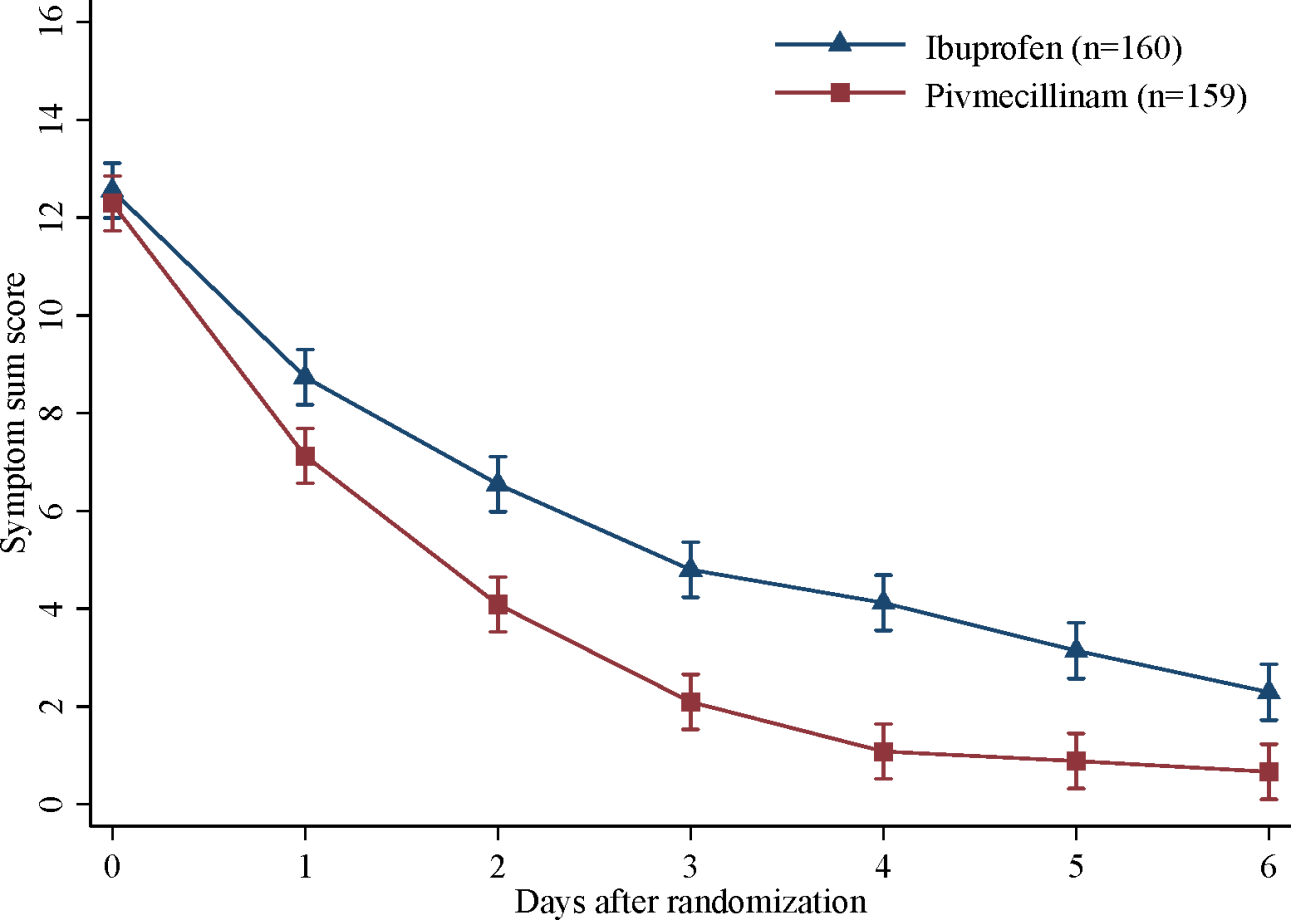
Estimated marginal mean symptom sum score (dysuria, urinary urgency, and urinary frequency) with 95% CI from the linear mixed model by treatment group (range 0–18).

Throughout the first week of follow-up, the ibuprofen group had a higher symptom burden than the pivmecillinam group. At day 6, the mean symptom sum score was 2.3 for the ibuprofen group and 0.7 for the pivmecillinam group, giving an estimated difference of 1.6 (95% CI 0.8 to 2.4) (Fig 3). Clinical measures were generally in favor of pivmecillinam, both in the FAS (Table 2) and the per protocol set (S1 Table).

Within 2 weeks, 41.4% of the patients in the ibuprofen group had a second consultation and were prescribed antibiotic treatment versus 9.6% in the pivmecillinam group. In the ibuprofen group, 47.0% had 1 or more secondary treatments with antibiotics within 4 weeks, versus 11.2% in the pivmecillinam group. For the purpose of these analyses, where we did not have information on whether the patient had a second consultation, we assumed she had received secondary treatment with antibiotics. Robustness analyses assuming no secondary treatment with antibiotics where we had missing data gave highly similar results with a treatment difference in the FAS of −0.36 (95% CI −0.45 to −0.28).

### Microbiology

At inclusion, there was significant bacterial growth in 121 (67.2%) of the cultures in the ibuprofen group and 113 (63.8%) in the pivmecillinam group. After treatment, the numbers were 43 (27.9%) in the ibuprofen group and 16 (10.4%) in the pivmecillinam group (*p* < 0.001) (Table 2). In the analyses, we assumed that missing cultures were positive.

In the ibuprofen group, 53% of the patients with baseline growth of *E*. *coli* and 53% of the patients with baseline growth of *S*. *saprophyticus* returned and received antibiotic treatment within 4 weeks. In the pivmecillinam group, the corresponding numbers were 13% for *E*. *coli* and 25% for *S*. *saprophyticus* (S2 Table). Persistent bacteriuria with the same microbe was found in 23 patients in the ibuprofen group and 4 patients in the pivmecillinam group (S3 Table).

### Subgroup analyses

**Fig 4:**
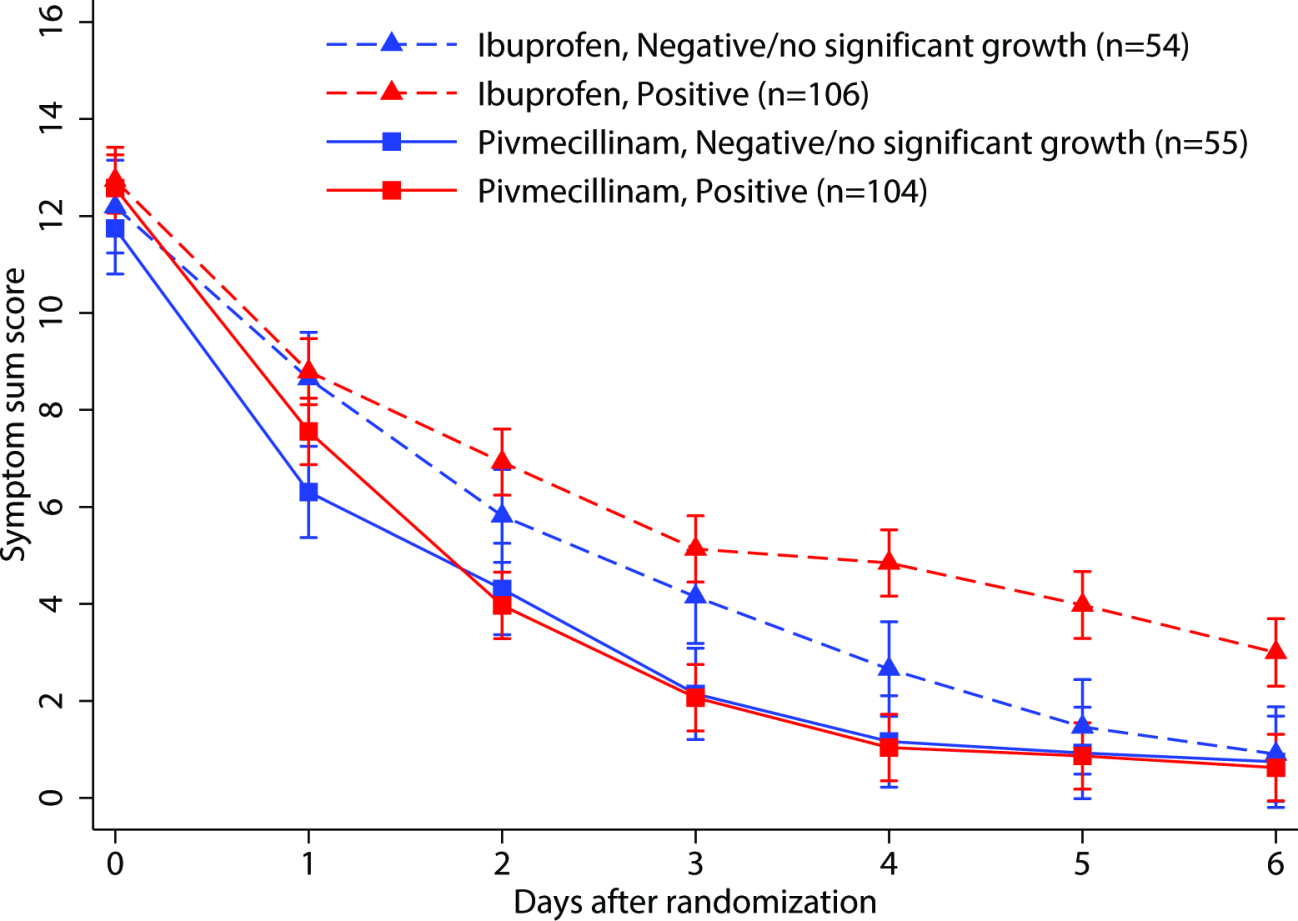
Estimated marginal mean symptom sum score with 95% CI from the linear mixed model by treatment group classified by urine culture positive or negative/no significant growth at inclusion.

For patients with a positive urine culture at baseline, pivmecillinam treatment led to a significantly lower symptom score at day 6 compared with ibuprofen treatment. For patients with a negative baseline urine culture, the symptom score at day 6 was similar in the 2 treatment groups (Fig 4). In the culture negative subgroup, 52% in the ibuprofen group and 71% in the pivmecillinam group felt cured by day 4, with an adjusted risk difference of 19% (95% CI 2% to 36%). In the culture positive subgroup, the numbers were 32% and 75%, respectively, with an adjusted risk difference of 43% (95% CI 31% to 55%). The interaction between treatment and urine culture on the proportion of patients who felt cured by day 4 was significant, indicating that there is a difference in the effect of pivmecillinam between patients with negative versus positive urine cultures.

As preplanned, we looked at the symptom burden for the patients with a positive baseline urine culture and grouped them by susceptibility to pivmecillinam. The difference in symptom burden between the ibuprofen group and the pivmecillinam group was numerically, but not significantly, bigger in the non-susceptible subgroup than in the susceptible subgroup (*p* = 0.22 at day 6) (S2 Fig).

Comparing the subgroup with recurrent UTIs (i.e., 3 or more UTIs during the previous 12 months) to the subgroup with 0–2 UTIs within the last 12 months, the highest symptom burden over 6 days was observed in the recurrent UTI patients treated with ibuprofen (S3 Fig). The difference in symptom burden between the treatment groups was bigger for those with recurrent UTIs, but significant in both subgroups.

We also compared the symptom burden for the patients with significant growth of *E*. *coli* in the baseline urine culture to those with significant growth of any other uropathogen. The difference in symptom burden between the treatment groups was bigger in the other uropathogen subgroup, but significant in both (S4 Fig).

### Safety

There were 7 SAEs during the trial, 1 in the pivmecillinam group and 6 in the ibuprofen group. A detailed description is given in Table 3. During the trial, 5 patients developed a febrile UTI and 7 patients developed pyelonephritis, all initially treated with ibuprofen. The 5 patients with febrile UTI were treated with antibiotics according to protocol and recovered fully as outpatients. Two of the patients diagnosed with pyelonephritis were treated as outpatients, and 5 were hospitalized. The 5 hospitalizations were all classified as SAEs and reported to the Norwegian Medicines Agency according to GCP. Seven cases of pyelonephritis in the ibuprofen group constitutes 3.9% of these patients, giving a number needed to harm (NNH) of 26 (95% CI 13 to 103). Five of these were hospitalized, with a NNH of 36 (95% CI NNH 16 to 792). As for relapses, there were 83 (46%) in the ibuprofen group versus 18 (10%) in the pivmecillinam group, with a NNH of 3 (95% CI 2 to 4).

**Table 3:** Details of serious adverse events: Intention to treat population of women with uncomplicated urinary tract infection randomized to either ibuprofen or pivmecillinam.

Diagnosis	Age (years)	Onset*	Brief history	Urine culture**	Trial drug
Alcoholic withdrawal	43	4	Admitted to hospital due to withdrawal symptoms after heavy drinking. Not related to study drug.	*E*. *coli*	Pivmecillinam; completed treatment
Abdominal pain	37	5	Treated with amoxicillin after 5 days. Admitted to hospital with abdominal pain after 9 days, with CRP 102 mg/l; discharged after 24 hours, with no antibiotics. Full recovery.	*E*. *coli*	Ibuprofen; completed treatment
Pyelonephritis	41	2	Treated with pivmecillinam after 2 days. Admitted to the hospital on day 3, with CRP 208 mg/l; received 3 doses of pivmecillinam i.v.; discharged after 24 hours with pivmecillinam for 10 days. Full recovery.	*E*. *coli*	Ibuprofen; took 6/9 capsules
Pyelonephritis	20	4	Got prescription for trimethoprim after 4 days; did not take it; felt worse after 7 days, with CRP 102 mg/l; admitted to hospital for 3 days; treated with gentamycin i.v.; discharged with pivmecillinam. Full recovery.	*E*. *coli*	Ibuprofen; completed treatment
Pyelonephritis	54	1	Treated with trimethoprim after 1 day. Returned on day 3 with flank pain and CRP 46 mg/l. Diagnosed with kidney stone; continued treatment with trimethoprim. Admitted to hospital after 5 days, with CRP 117 mg/l; treated with cefotaxime i.v. for 3 days; discharged with ciprofloxacin for 10 days. Full recovery.	*E*. *coli*	Ibuprofen; took 3/9 capsules
Pyelonephritis	18	7	Treated with pivmecillinam after 7 days. Switched to amoxicillin by family physician on day 8; admitted to hospital by study doctor same evening, with CRP 116 mg/l; treated with ampicillin and gentamicin i.v. for 24 hours; discharged with trimethoprim-sulfamethoxazole for 7 days. Full recovery.	*E*. *coli*	Ibuprofen; completed treatment
Pyelonephritis	27	5	Admitted to hospital in England after 5 days; received treatment with unknown antibiotics i.v.; discharged after 10 days, with full recovery after 13 days.	No significant growth	Ibuprofen; completed treatment

*Number of days from inclusion.

**Baseline urine culture.

CRP, C-reactive protein.

There was no need for unblinding during the trial.

Among the 12 patients who developed a febrile UTI or pyelonephritis, 10 patients had a positive urine culture at baseline: 8 showed growth of *E*. *coli*, and 2 showed growth of *S*. *saprophyticus*. For the 2 other patients, 1 had non-significant growth of mixed bacterial flora and 1 had a negative culture, but a second culture taken after 5 days showed growth of *E*. *coli*. For details on the patients who developed a febrile UTI or pyelonephritis, but were not classified as having SAEs, see S4 Table. There were a few more self-reported adverse events in the pivmecillinam group than in the ibuprofen group, but the differences were not statistically significant (*p* = 0.68) (Table 4).

**Table 4:** Details on self-reported adverse events: Intention to treat population of women with uncomplicated urinary tract infection randomized to either ibuprofen or pivmecillinam.

Classification (ICD-10)	Ibuprofen (*n =* 181)	Pivmecillinam (*n =* 178)	*p*-Value
**No adverse events, *n* (%)**	127 (70.2%)	122 (68.5%)	0.68
**Diseases of the digestive system (K00–K93)—such as blisters in mouth**	1	0	
**Diseases of the musculoskeletal system and connective tissue (M00–M99)—such as back pain**	0	1	
**Diseases of the genitourinary system (N00–N99)—such as vaginal thrush and local infection**	4	3	
**Symptoms, signs, and abnormal clinical and laboratory findings not elsewhere classified (R00–R99)**			
Symptoms and signs involving the circulatory and respiratory systems (R00–R09)—such as unspecified chest pain	0	1	
Symptoms and signs involving the digestive system and abdomen (R10–R19)—such as heartburn, nausea, abdominal pain, and change in bowel habit	20	27	
Symptoms and signs involving cognition, perception, emotional state, and behavior (R50–R69)—such as headache, fatigue, and dizziness	15	17	
**No information**	22	18	
**Total number of adverse events/patients with adverse events**	40/32	49/38	

## Discussion

### Principal findings

In this study, we were unable to show that ibuprofen was non-inferior to pivmecillinam for treating uncomplicated UTI. Reducing the use of antibiotics came at the cost of stronger symptom burden, longer duration of symptoms, and more complications.

In the pivmecillinam group, there were no significant differences in symptom burden and time to resolution of symptoms between those with a positive and negative urine culture.

In the ibuprofen group, the patients with a positive culture had a higher symptom burden and it took longer to achieve symptom resolution than for those with a negative culture. Furthermore, 10 of the 12 patients with complications had a positive urine culture at baseline showing growth of a primary pathogen: 8 *E*. *coli* and 2 *S*. *saprophyticus*. This indicates that patients with a positive culture are more likely to benefit from antibiotic treatment and that treating these patients could prevent unwanted complications.

However, nearly half of the patients in the ibuprofen group with a positive urine culture at baseline recovered without antibiotics (S2 Table). About one-third of the patients in the ibuprofen group with negative urine culture at baseline needed antibiotic treatment to recover. A minority (5%) of patients in the pivmecillinam group with negative baseline urine culture needed a second treatment with antibiotics to recover. This shows that distinguishing those patients who are likely to benefit from symptomatic treatment alone from those who will need antibiotic treatment, particularly at baseline, is still a challenge. We will conduct exploratory analyses to see if we can identify predictors at baseline that could help healthcare providers decide which patients should be recommended symptomatic relief, and maybe a wait-and-see prescription for antibiotics, and which patients should receive immediate treatment with antibiotics.

### Strengths and weaknesses of the study

The strengths of our study were that the blinding was well performed, we reached our calculated sample size, and the dropout rate was less than 10%. Our participants were recruited from unselected patients presenting with UTI symptoms. We recruited patients from both general practices and outpatient clinics; thus, we have a quite diverse population.

A weakness was the extensive list of exclusion criteria, eliminating almost half of the patients presenting with symptoms of an uncomplicated UTI. Many eligible patients were not asked to participate, mainly due to the heavy work load at the AEOCs. If the pressure of incoming patients was too high, the nurses did not feel they could prioritize enrolling patients for the trial, and sometimes the nurses had not yet received the proper training to enroll patients. Furthermore, we did not include symptom burden in the questionnaire at inclusion; hence, we do not know the degree of symptoms for those who declined to participate. This might make our results less generalizable. A few patients lost the diary and equipment to send the second urine culture; hence, we had to send new diaries and equipment by mail, and in some instances this led to a delay of up to several weeks in collecting data. Filling out the diary retrospectively might provide less reliable data.

As the distinction between febrile UTI and pyelonephritis was not defined in the protocol, we went through the medical records and made the classification based on the doctors’ description of the patients’ symptoms and CRP value. The classification was done prior to unblinding the treatment allocation and thus should not have introduced bias.

All patients were allowed to take paracetamol as additional pain medication if needed. The patients did not report this, so we do not know how many actually took additional paracetamol. If the patients in the ibuprofen group took additional paracetamol, it could possibly have masked symptomatic progression of upper UTI.

### Comparison with existing studies and further research questions

We chose to use ibuprofen over other NSAIDs because of its relatively low rate of severe adverse effects. The dosage used was lower than the maximum recommended daily intake. We chose to use an NSAID instead of paracetamol because of its greater anti-inflammatory effect, which led us to believe it would provide better pain relief. Using a placebo as a second comparator was discussed, but given the number of placebo trials proving inferiority to antibiotics, we felt this would be unethical. We chose pivmecillinam because it is a narrow spectrum antibiotic and one of the first choice antibiotics for treating uncomplicated UTI in Scandinavia [23]. It has selective activity against Gram-negative bacteria, especially *E*. *coli*, a low resistance-driving effect, and limited side effects [24,25]. We believe that pivmecillinam could be considered as an alternative first choice agent in other countries.

In our study, using worst case imputation for missing data, 53% of the patients in the ibuprofen group recovered without antibiotics, compared to 67% in the study conducted by Gagyor et al. [14] and 38% in the trial by Kronenberg et al. [15].

The pivmecillinam group in our study recovered more quickly and had fewer complications than the fosfomycin group in the trial by Gagyor et al. This could indicate that a 3-day treatment with pivmecillinam is more effective than a single dose of fosfomycin. In the trial by Kronenberg et al., they defined symptom resolution as a symptom score of less than 2, whereas in our trial and the trial by Gagyor et al., it was defined as a symptom score of 0. If we compare the number of patients with a symptom score of 0 by day 4, the pivmecillinam group recovered more quickly than the patients treated with norfloxacin. Even though the 3 study populations may differ somewhat, they have very similar inclusion and exclusion criteria and the study drugs are similar. Despite the discrepancies, we believe it is relevant to compare the different treatment groups from the 3 trials for the purpose of discussion.

Our ibuprofen group had a higher symptom burden, longer duration of symptoms, and more complications than the ibuprofen group in the trial by Gagyor et al. It could be questioned whether it is safer to treat patients presenting with an uncomplicated UTI with 400 mg rather than 600 mg of ibuprofen. The trial by Gagyor et al. had 5 cases of pyelonephritis in the ibuprofen group and 1 in the fosfomycin group. The trial by Kronenberg et al. had 6 cases (5%) of pyelonephritis, all among the patients initially treated with diclofenac. We had 7 cases of pyelonephritis, all in the ibuprofen group. The rate of pyelonephritis in the ibuprofen group was 3.9%, and even though the number of patients was quite small, the percentage is high compared to what was found in a meta-analysis of randomized controlled trials comparing placebo with antibiotics, where the incidence of pyelonephritis in the placebo group ranged from 0.4% to 2.6% [12]. Based on the results of our study, if we treated 3 patients with ibuprofen, 1 of them would need to be retreated with antibiotics, compared to if all patients were treated with pivmecillinam (NNH = 3), so the risk of needing retreatment with antibiotics is relatively high in the ibuprofen group. With NNH = 26 for developing pyelonephritis and 36 for needing hospitalization, the risk of getting a potentially serious infection was elevated in the ibuprofen group, but still relatively low. Several studies have reported more frequent and more serious complications in patients receiving ibuprofen or other NSAIDs [14,15,26–29]. Ibuprofen may have a local immune-compromising effect and thus increase the risk for upper UTI [30]. This might also be the case for diclofenac. This raises the question of whether other drugs providing symptom relief, such as paracetamol, might be better and safer than NSAIDs.

The German group identified a moderate to severe degree of urinary urgency/frequency and a positive urinary dipstick for erythrocytes, leucocytes, and nitrite as predictors for needing subsequent antibiotic treatment [31]. In the trial by Kronenberg et al., they found that baseline CRP levels >10 mg/l were positively correlated with developing pyelonephritis [15]. Both the Swiss group and our group are contributing to a meta-analysis of individual data from several trials comparing antibiotic treatment to either placebo or symptomatic treatment. This collaborative work was initiated by the German group.

In our study, more than half of the women treated with ibuprofen achieved symptomatic cure without subsequent antibiotic treatment. This is in line with the findings of Little et al., who showed that a wait-and-see strategy for women with uncomplicated UTI reduced the use of antibiotics in a safe manner [32]. With shared decision-making, some women might accept and benefit from initial symptomatic treatment and a delayed prescription for antibiotics. This could contribute to reducing antibiotic overuse.

## Conclusion

More than half of the women treated with ibuprofen achieved symptom resolution without any additional treatment. Initial treatment with ibuprofen could reduce unnecessary use of antibiotics in this group. However, until we can identify those women in need of antibiotic treatment to prevent complications, we cannot recommend ibuprofen alone to women with uncomplicated UTIs.

## Supporting information

S1 Appendix Statistical analysis plan. (PDF)

S1 CONSORT Checklist (DOC)

S1 Data (ZIP)

S1 Fig Questionnaire with inclusion and exclusion criteria for participation in the trial of ibuprofen versus pivmecillinam for women with uncomplicated UTI. (TIF)

S2 Fig Estimated marginal mean symptom sum score with 95% CI from the linear mixed model by treatment group for those patients with significant bacterial growth at baseline classified by susceptible versus non-susceptible to pivmecillinam. (TIF)

S3 Fig Estimated marginal mean symptom sum score with 95% CI from the linear mixed model by treatment group classified by number of previous UTIs within last 12 months before inclusion: 0–2 episodes versus ≥3 episodes. (TIF)

S4 Fig Estimated marginal mean symptom sum score with 95% CI from the linear mixed model by treatment group classified by significant bacterial growth of *E*. *coli* versus other uropathogen at baseline. (TIF)

S1 Protocol (PDF)

S1 Table Summary of primary and key secondary outcomes in women with uncomplicated UTI randomized to either ibuprofen or pivmecillinam: Per protocol population. Figures are number of women (percentage) unless stated otherwise.
(DOCX)

S2 Table Need for secondary treatment with antibiotics by initial infecting organism: Intention to treat population of women with uncomplicated UTI randomized to either ibuprofen or pivmecillinam. Figures are proportions (percentage) unless stated otherwise.
(DOCX)

S3 Table Baseline urine culture versus second urine culture: Intention to treat population of women with uncomplicated UTI randomized to either ibuprofen or pivmecillinam. (DOCX)

S4 Table Details of patients with febrile UTI or pyelonephritis not classified as serious adverse events: Intention to treat population of women with uncomplicated UTI randomized to either ibuprofen or pivmecillinam. (DOCX)

## Abbreviations

AEOC: accident and emergency outpatient clinic
CRP: C-reactive protein
FAS: full analysis set
GCP: good clinical practice
GP: general practitioner
NNH: number needed to harm
NSAID: nonsteroidal anti-inflammatory drug
SAE: serious adverse event
SAP: statistical analysis plan
UTI: urinary tract infection
