# Supplementary material for: Ibuprofen versus pivmecillinam for uncomplicated urinary tract infection in women—A double-blind, randomized non-inferiority trial
Source: PLoS Med. 2018 May 15;15(5):e1002569. doi: 10.1371/journal.pmed.1002569 (PMC5953442; doi:10.1371/journal.pmed.1002569)
Supplement: S1 Appendix — (PDF) [file pmed.1002569.s001.pdf]

IMUTI

Statistical Analysis Plan

A randomized, double-blind trial to compare ibuprofen versus mecillinam in the treatment of uncomplicated cystitis in adult, non-pregnant women.

The IMUTI study

Final Protocol Version 4.0: 19 March 2013

EudraCTnr: 2012-002776-14.

ClinicalTrials.gov: NCT01849926.

SAP Version 1.0 Final

Date: 08 June 2017

Approval Page

Biostatistician Oslo University Hospital

Signature ____

Inge Christoffer Olsen, PhD

Date: __8 June 2017_____________________________________

Principal investigator/Project leader

Signature ____

Morten Lindbaek, MD PhD Prof.

Date ____8 June 2017____

Abbreviations

AB	Antibiotics
AE	Adverse Event
ARD	Adjusted Risk Difference
ARR	Adjusted Relative Risk
AUC	Area under the curve
CI	Confidence Interval
CRP	C-reactive protein
CSR	Clinical Study Report
EOT	End of Treatment
FAS	Full analysis Set
GCP	Good Clinical Practice
GI	Gastrointestinal
HR	Hazard Ratio
ICF	Informed Consent Form
ISF	Investigator Site Files
ITT	Intention to treat
KM	Kaplan Meier
NK	Not Known
NRS	Numeric rating scale
PH	Proportional Hazards
PP	Per Protocol
PRO	Patient reported outcome
PT	Preferred Term
SAE	Serious Adverse Event
SD	Standard deviation
SE	Standard Error
SOP	Standard Operating Procedure
SUSAR	Suspected Unexpected Serious Adverse Reaction
TEAE	Treatment Emerging Adverse Events
TID	Ter In Die (Three Times a Day)
TFL	Tables Figures Listings
TMF	Trial master file
UTI	Urinary Tract Infection

TABLE OF CONTENTS

1. Amendments from previous version ... 5
2. INTRODUCTION ... 6
2.1 Study Objectives ... 6
2.1.1 Primary Objective ... 6
2.1.2 Secondary Objectives ... 6
2.2 Study Design ... 6
3. Hypotheses and decision rules ... 7
3.1 Statistical Hypotheses ... 7
3.2 Statistical Decision Rule ... 7
4. ANALYSIS SETS ... 7
4.1 Enrolled ... 7
4.2 Full Analysis Set ... 7
4.3 Safety Analysis Set ... 8
4.4 Per Protocol Analysis Set ... 8
4.5 Treatment Misallocation ... 8
4.6 Protocol Deviation ... 8
4.6.1 Deviations to inclusion and/or exclusion criteria ... 8
4.6.2 Deviations assessed Post-randomization ... 8
5. DEFINITIONS AND DERIVED VARIABLES ... 8
5.1 Change from baseline ... 9
5.2 Symptoms score ... 9
5.3 Urine cultures ... 9
5.4 Inflammation parameters ... 9
5.5 Febrile Urinary Tract Infection (UTI) and Pyelonephritis ... 9
5.6 Secondary treatment with antibiotics ... 9
5.7 Patient reported outcomes ... 10
5.8 Serious adverse events ... 10
5.9 Other calculations ... 10
6. Efficacy Endpoints ... 10
6.1 Primary endpoint ... 10
6.2 Secondary endpoints ... 10
6.2.1 Efficacy endpoints ... 10
6.2.2 Safety parameters ... 10
7. STATISTICAL METHODOLOGY ... 11
7.1 Statistical and Analytical Issues ... 11
7.1.1 Statistical Methods ... 11
7.1.2 Pooling of Investigator Sites ... 13
7.1.3 Determination of Sample Size ... 13
7.1.4 Timing of Main Analysis ... 13
7.2 Patient Characteristics ... 13
7.2.1 Patient Disposition ... 13
7.2.2 Background and Demographic Characteristics ... 13
7.2.3 Treatment Compliance ... 13
7.2.4 Exploratory Analysis ... 13
8. Safety Analysis ... 14
8.1.1 Adverse Events ... 14
8.2 Interim Analyses ... 14
8.2.1 Independent Data Monitoring Committee (IDMC) ... 14
9. REFERENCES ... 14
10. LIST OF PLANNED TABLES, FIGURES AND LISTINGS ... 14
10.1 Data Tables ... 14
10.2 Data Listings ... 15
10.3 Data Figures ... 15

1. Amendments from previous version

Not applicable

2. INTRODUCTION

This document describes the planned data summaries and statistical analyses to be performed for the Clinical Trial Protocol IMUTI: A randomized, double-blind, parallel-group study to evaluate the safety and efficacy of ibuprofen versus mecillinam in the treatment of uncomplicated cystitis in adult, non-pregnant women: The IMUTI study. It is intended to supplement the study protocol, which contains details regarding the objectives and design of the study.

2.1 Study Objectives

2.1.1 Primary Objective

The primary hypothesis of this study is that symptomatic treatment with ibuprofen is not inferior to mecillinam in the treatment of uncomplicated cystitis in otherwise healthy, adult, non-pregnant women.

2.1.2 Secondary Objectives

The secondary objectives of this study are:

- To compare the efficacy of treatment with ibuprofen versus treatment with mecillinam with regards to symptom relief and bacteriological cure in patients with uncomplicated cystitis/UTI
- To assess the safety of treatment with ibuprofen versus treatment with mecillinam with regards to number of secondary doctor's consultations leading to an antibiotic prescription, development of an upper UTI within the 28 days of follow-up
- To assess the number and type of self-reported adverse events within the first seven days as recorder in the patient diary
- To assess the serious adverse events within the 28 days of follow-up

2.2 Study Design

The IMUTI study is designed as a randomized, double blind, controlled, parallel-group, multi-centre, multi-country, non-inferiority comparative phase IV study.

Any non-pregnant female patient between 18 and 60 years of age presenting with symptoms of an uncomplicated cystitis/UTI is a potential study patient. Eligible patients with informed consent will be randomized to either treatment with ibuprofen 600 mg TID for three days or treatment with mecillinam 200 mg TID for three days.

In order to balance the patient characteristics on the two treatment arms, randomisation will be stratified according to study site. However, due to anticipated low patient numbers, patients in Denmark and Sweden will be stratified according to country, while in Norway stratification will be performed according to study site. Randomisation will be performed using computer, with random block size of 2, 4 and 6. Approximately 400 patients are to be accrued in this study. The main analysis will be performed after all patients have completed the 28 days of follow-up, and the primary measure of efficacy is proportion of patients who felt cured by day four after randomisation.

3. Hypotheses and decision rules

3.1 Statistical Hypotheses

This study is designed to establish the non-inferiority of ibuprofen compared to mecillinam treatment regarding symptomatic relief four days after treatment initiation.

The null hypothesis is that ibuprofen treatment is inferior to mecillinam treatment with regards to the proportion of patients who felt cured four days after treatment initiation by a 10% inferiority margin. The alternative hypothesis is that ibuprofen treatment is non-inferior to mecillinam treatment with regards to the proportion of patients who felt cured four days after treatment initiation by a 10% inferiority margin.

3.2 Statistical Decision Rule

This protocol is designed to address a single primary endpoint. Non-inferiority is claimed if the null hypothesis is rejected on the significance level (alpha) of 0.05 (one-sided). That is, if the limit of the one-sided 95% confidence interval for the treatment difference is less than 10% (see Figure 3.1).

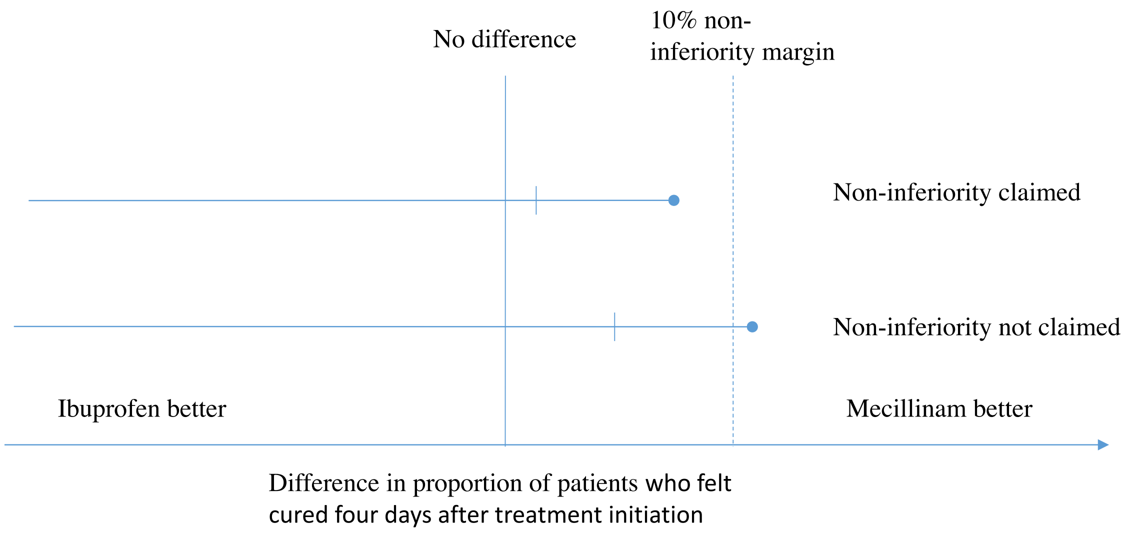

Figure 3.1 Illustration of Statistical Decision Rule (bars indicate 95% one-sided confidence limits)

4. ANALYSIS SETS

4.1 Enrolled

The Enrolled set will include all patients who have provided informed consent and have been included into the study data base.

4.2 Full Analysis Set

The Full Analysis Set (FAS) will be defined as all patients randomly assigned to a treatment group having at least one efficacy assessment after randomisation.

4.3 Safety Analysis Set

The Safety Set will include all randomised patients with at least one safety assessment after randomisation.

4.4 Per Protocol Analysis Set

The Per Protocol Analysis Set (PPS) will include all randomised patients meeting the study eligibility criteria, at least one efficacy assessment after randomisation and with a treatment compliance rate of at least 80%.

4.5 Treatment Misallocation

- If patients were randomized but not treated: patient will appear on the patient evaluation table as randomized but not treated; this is the extent of how much the patient will be reported.
- treated but not randomized: then by definition the patient will be excluded from both the efficacy and safety analyses since randomized treatment is missing
- randomized but did not follow protocol treatment compliance according to allocation: then they will be reported under their randomized treatment group for all efficacy and safety analyses as part of the FAS and safety analyses, but omitted from the PPS

4.6 Protocol Deviation

The following sections describe any protocol deviations that relate to the statistical analyses and forms the requirement for exclusion from the PPS.

4.6.1 Deviations to inclusion and/or exclusion criteria

Any patient who enters the study when the inclusion or exclusion criteria would have prevented entry will be considered to have had a protocol deviation.

4.6.2 Deviations assessed Post-randomization

Only protocol deviations thought to affect the efficacy of the study treatment will be considered in the SAP. Patients will be considered to have a protocol deviation if they failed to fill in the patient diary according to protocol or did not take sufficient amount of treatment defined as at least 8 out of 9 capsules of study medication. The requirement of diary completeness is disregarded if there is information on remission by telephone contact.

5. DEFINITIONS AND DERIVED VARIABLES

For all clinically planned measures, visits should occur within a window of the scheduled visit. Since visits follows the treatment frequency, the target day and visits window is defined as:

Label	Target Day	Definition (Day window)
Screening	Day 0	Day 0
Baseline (Day 0)	Day 0 (Randomization)	Day 0
Day 1	Day 1	Target day
Day 2	Day 2	Target day
Day 3	Day 3	Target day
Day 4	Day 4	Target day
Day 5	Day 5	Target day
Day 6	Day 6	Target day
Day 14	Day 14	Target day ± 4 days
Day 28	Day 28	Target day ± 7 days

5.1 Change from baseline

Change from baseline (Δ) = time-point value - baseline value.
% change from baseline (%Δ) = [(time-point value – baseline value) / baseline value] *100%

5.2 Symptoms score

- Dysuria is evaluated on a 7-point Likert scale (0-6) with 0 indicating normal/no problem and 6 indicating as bad as it can be.
- Urinary urgency is evaluated on a 7-point Likert scale (0-6) with 0 indicating normal/no problem and 6 indicating as bad as it can be.
- Urinary frequency is evaluated on a 7-point Likert scale (0-6) with 0 indicating normal/no problem and 6 indicating as bad as it can be.
- Abdominal pain when not passing urine is evaluated on a 7-point Likert scale (0-6) with 0 indicating normal/no problem and 6 indicating as bad as it can be.
- Feeling unwell is evaluated on a 7-point Likert scale (0-6) with 0 indicating normal/no problem and 6 indicating as bad as it can be.
- Visible blood in urine is indicated by yes or no.
- Fever is indicated by yes or no.
- Adverse events/side effects are indicated by yes or no. If yes is indicated the type of side effect is described by the patient.
- "I feel cured" indicates that the patient feels completely cured of the urinary tract infection.
- Symptoms sum score will be calculated as the sum of the dysuria, urinary urgency and urinary frequency sub-scores.

5.3 Urine cultures

Urine samples are obtained and cultured at inclusion and after 14 days. If the patient comes back for a second doctor's consultation within the 28 days of follow-up a third urine culture might be registered. Significant bacteriuria and thereby a positive urine culture was defined according to current European guidelines for patients with symptoms of UTI as $\geq 10^3$ /mL for primary pathogens, $\geq 10^4$/mL for secondary pathogens and $\geq 10^5$/mL for doubtful pathogens. {Aspevall:2001hg}

5.4 Inflammation parameters

Inflammation parameters include the high sensitivity C-reactive protein (CRP) in mg/L. CRP is an objective criterion for degree of inflammation and disease severity in all infectious diseases. This parameter was not routinely measured, but if a patient came back for a second doctor's consultation within the 28 days of follow-up CRP was often measured.

5.5 Febrile Urinary Tract Infection (UTI) and Pyelonephritis

Patients with a secondary consultation were assessed for febrile urinary tract infection (UTI) and pyelonephritis. The assessments were done centrally based on the patient's diary, reports from the telephone contact and patient medical journal if available. We used the patients' symptoms as an indication on the severity of the UTI together with the CRP. If the patient reported fever and flank pain, with or without the continued presence of dysuria, urinary urgency and urinary frequency, and had CRP < 40 we classified them as having a febrile UTI. If they had CRP > 40 we classified the infection as pyelonephritis. If the patient had pyelonephritis and in need of hospitalisation the episode was also classified as a SAE.

5.6 Secondary treatment with antibiotics

A secondary prescribed treatment with antibiotics for UTI is reported during the telephone contact after 14 days and 28 days. Patients are regarded as having had a secondary treatment with antibiotics regardless of compliance.

5.7 Patient reported outcomes
There were no patient reported outcomes in this study

5.8 Serious adverse events
Adverse events are reported in the patient's diary. Any event leading to hospitalisation is regarded as a serious adverse event (SAE).

5.9 Other calculations
Age (years) = [(date of baseline – date of birth)/365.25].

Time of event = day of event – day of randomization

6. Efficacy Endpoints
6.1 Primary endpoint
The primary endpoint is the proportion of patients who felt cured by Day 4 as registered in the patient diary. Specifically, a patient is regarded as "felt cured" if they by any diary registration up to and including Day 4 indicated "I feel cured".

6.2 Secondary endpoints

6.2.1 Efficacy endpoints

Endpoint	Type
Felt cured	Time to event
Felt cured by day 7	Dichotomous
Felt cured by day 14	Dichotomous
Symptom scores	
Sum score	Continuous
Dysuria	Continuous
Urinary urgency	Continuous
Urinary frequency	Continuous
Visible haematuria	Dichotomous
Urine culture after 14 days	Dichotomous
Significant growth of primary pathogen	Dichotomous
Significant growth of primary or secondary pathogen	Dichotomous
Significant growth of primary, secondary or doubtful pathogen	Dichotomous
Secondary treatment with antibiotics	
Within 14 days after treatment start	Dichotomous
Between 14 and 28 days after treatment start	Dichotomous
Within 28 days after treatment start	Dichotomous
Febrile UTI or Pyelonephritis	Ordinal
SAE	Dichotomous

6.2.2 Safety parameters
Measures of safety will include the following:

- Self-reported adverse events (AEs)

- Patients developing a febrile UTI
- Patients developing a pyelonephritis
- Hospital admissions (SAEs)
- Suspected Unexpected Serious Adverse Reactions (SUSARs)

7. STATISTICAL METHODOLOGY

7.1 Statistical and Analytical Issues

7.1.1 Statistical Methods

The primary efficacy analyses will be based on the Full Analysis Set (FAS). Secondary efficacy analyses will be based both on the FAS and the Per Protocol Set (PPS) As there is only one identified primary analysis, there will be no adjustments for multiple testing in the secondary analyses.

All categorical (binary and ordinal) data will be summarised using frequency counts and percentages of patient incidence. Percentages will be calculated using the study population (FAS); any exceptions to this will be highlighted in the table footnote. The continuous variables will be summarised using number of patients (N), mean, standard deviation (SD), median, 25/75 percentile and range (minimum/maximum). In general, minimum and maximum will be presented to the same degree of precision as data is recorded, with mean and median having 1 additional place after the decimal and standard deviation having 2 additional places after the decimal. Percentages less than 100 will be displayed to 1 place after the decimal, where space permits.

When random numbers are warranted for inference (such as for bootstrapping and multiple imputation), the seed will be set to the date the statistical analysis plan is signed-off (in the format yyyymmdd).

For the primary analysis, the point estimate will be presented with a one-sided 95% confidence limit to assess the non-inferiority hypothesis. Other efficacy analyses will be presented by the size (point estimate) of the difference between the treatments and the associated two-sided 95% confidence interval. P-values are generally not presented as this is a non-inferiority trial, and presentation of the p-value could be misinterpreted.

All statistical analyses will be done in Stata v14 (StataCorp. 2015. *Stata Statistical Software: Release 14*. College Station, TX, USA).

7.1.1.1 Analyses of primary endpoint

The primary endpoint (proportion of patients who felt cured by day four) will be analysed using logistic regression with treatment as fixed effect, adjusted for randomisation strata providing estimates (by the delta method) of adjusted risk difference (ARD) and adjusted relative risk (ARR) for the treatment effect.

The primary analysis will be performed on the primary analysis set, (the Full Analysis Set), see section 4.4 for formal definition.

Missing data imputation and sensitivity analyses

When patient assessment of disease state is missing in the diary, information on "feeling cured" obtained from the follow-up telephone contact will be used when available. When no information on primary endpoint exist, worst outcome (not feeling cured by Day 4) will be imputed. A best outcome imputation (feeling cured by Day 4) will be performed as a robustness analysis.

Blinded pre-lock assessment of the primary endpoint showed complete registration for the Full Analysis Set. There will therefore be no imputation for the primary endpoint in this analysis set.

There will be no missing data for the primary endpoint in the Per Protocol analysis set.

7.1.1.2 Analysis secondary dichotomous endpoints

Analysis of single observed dichotomous endpoints will be performed using logistic regression adjusted for randomisation strata providing estimates of adjusted risk difference (ARD) and adjusted relative risk (ARR) (by the delta method) for the treatment effect.

Analysis of repeated dichotomous endpoints will be performed using mixed effects logistic regression adjusted for randomisation strata providing estimates of risk by day after treatment.

Missing data and imputation

When no information on status of the dichotomous endpoint exist, worst outcome imputation will be performed. The exception is SAE, where no information is regarded as no SAE.

7.1.1.3 Analysis of secondary continuous variables

Analyses of continuous endpoints will include linear mixed models with patient-specific random intercept and treatment, time, treatment-time interaction and randomisation strata.

Missing data and imputation

The analysis of continuous variables will be performed on the PPS and FAS. There will be no imputation of missing values as the mixed model provides unbiased estimates under the assumption of missing at random observations.

7.1.1.4 Analyses of time to event endpoints

Time to event endpoints (see section 6.2.1) will be analysed using a Cox regression model adjusted for randomisation strata. Time to event will be censored at study end (Day 14). Estimates of the hazard ratio will be presented in addition to Kaplan-Meier plots.

Missing data and imputation

For the time to event analysis, patients with incomplete information will be censored on the last day of information. The analysis of time to event endpoints will be analysed on the FAS and PPS. Patients with no information on time to event will be left censored, i.e. not included into the analysis. Robustness analysis will be performed where missing information on time to event will be imputed with worst outcome (censored at day 14).

7.1.1.5 Analyses of ordinal endpoints

Ordinal endpoints (febrile urinary tract infection or pyelonephritis) (see section 6.2.1) will be tabulated and analysed using Fisher's exact test of the 2x3 table due to low number of events.

Missing data and imputation

The analysis of ordinal variables will be performed on the PPS and FAS. Missing information will be imputed using best case, as no information on febrile urinary tract infection or pyelonephritis will be interpreted as no complication.

7.1.2 Pooling of Investigator Sites

The Norwegian study sites will remain study sites in the analyses, while the Swedish and Danish study sites will be pooled according to country due to several sites with few recruited patients.

7.1.3 Determination of Sample Size

We assume that 85% in the mecillinam group will feel completely cured after four days and consider a 10% absolute reduction to 75% in the ibuprofen group as a maximum relevant difference when holding that ibuprofen is non inferior to mecillinam. With alpha 0,05 and 1-beta of 80% we estimate that we need about 150 patients in each group. To add up for drop outs and withdrawals we aim at recruiting 200 in each group. The 85% assumption is based on a study on uncomplicated cystitis at the Oslo Accident and Emergency Outpatient Clinic where 85% of patients felt cured after four days.{Bollestad:2015cg}

7.1.4 Timing of Main Analysis

The main analysis is planned when all patients have concluded follow-up, all data have been entered, verified and validated and the database has been locked.

7.2 Patient Characteristics

7.2.1 Patient Disposition

The disposition of all patients will be listed and summarised by treatment arm. The number and percentage of patients who are randomised, received any study treatment, prematurely discontinued from treatment and lost to follow-up will be summarised.

The number and percentage of patients will be categorized by the reason(s) for

1) Discontinued from treatment: This is when a patient did not complete the treatment according to study protocol. Reasons can be: Lack of efficacy, Remission, adverse events, and other.
2) Lost to follow-up: This is when the patient withdraws or is withdrawn from the study, or failed to complete the diary or respond to telephone contact. Reasons can be: patient withdrawal of informed consent, adverse event, lost to follow-up, death, investigator decision or other.

7.2.2 Background and Demographic Characteristics

Patient demographics and baseline characteristics will be summarised both for the PPS and FAS.

Patient demographics and baseline characteristics will be summarised by randomised treatment arm and overall using descriptive statistics (N, mean, standard deviation, median, 25/75 percentiles, minimum, and maximum) for continuous variables, and number and percentages of patients for categorical variables. The patient demographics and baseline characteristics to be summarised include age in years and symptom duration, symptom score, urinary dipstick, recurrent UTI and urine culture.

7.2.3 Treatment Compliance

Data summarizing the proportions of patients complying with the treatment regimen according to protocol (number of self-reported capsules taken) will be presented by treatment arm.

7.2.4 Exploratory Analysis

Subgroup analysis will be performed for the primary and symptom sum score endpoints according to status of significant growth of primary, secondary and doubtful pathogens in the baseline urine culture

as well as susceptibility to mecillinam in the subpopulation of patients with significant growth of any pathogens. Subgroup analysis will be done using same model as for the main analyses, but with a subgroup interaction term. The subgroup analysis will be performed on the full analysis set only. Patients with missing baseline cultures will be regarded as having no pathogen growth.

Predictor analysis of baseline variables on efficacy endpoints will be performed.

8. Safety Analysis

General safety evaluations will be based on the incidence and type of AEs. Safety variables will be tabulated and presented for all patients in the safety set.

8.1.1 Adverse Events

Self-reported adverse events will be coded using ICD, version 10. Serious adverse events are any event that leads to hospitalisation within the study period, that is on or after the day of randomisation or within the 28 days of follow-up.

The number (%) of subjects with any AEs will be summarised by treatment group. SAEs will be detailed and presented in a separate table.

8.2 Interim Analyses

There are no planned interim analyses for efficacy.

8.2.1 Independent Data Monitoring Committee (IDMC)

No analyses have been made for the IDMC, and will not be reported in the clinical study report.

9. REFERENCES

10. LIST OF PLANNED TABLES, FIGURES AND LISTINGS

This section contains lists of all the summary tables, figures and patient data listings for this study.

10.1 Data Tables

Data tables will be configured according to publication requirements.

Table 1. Baseline data from ITT population (Full dataset).
Baseline characteristics for women with uncomplicated urinary tract infection randomised to iboprofen or mecillinam. Figures are number of women (percentage) unless stated otherwise.

Table 2. Summary of outcomes.
Summary of primary and key secondary outcomes in women with uncomplicated urinary tract infection randomised to iboprofen or mecillinam. Figures are number of women (percentage) unless stated otherwise.

Table 3. Duration of symptoms.

Table 4. Baseline data from subgroup populations/datasets.

Table 5. Details on adverse events.

Table 6. Details of SAE.

10.2 Data Listings

Data listings will be provided as needed.

10.3 Data Figures

Data figures will be configured according to publication requirements.

Figure 1. Flowchart.
Flow of participants through trial.

Figure 2. Total proportion of patients who felt well by randomisation group within day 4 and within day 7.

Figure 3. Total number of prescriptions/new treatments by randomisation group on days 0-28.

Figure 4. Symptom sum score for dysuria, urinary frequency and urinary urgency on days 0-7 by randomisation group.

Figure 5. Symptom sum score for women with negative urine cultures by randomisation group on days 0-7.

Figure 6. Symptom sum score for women with positive urine cultures by randomisation group on days 0-7.

Figure 8. Symptom score on day 0-7 for women in mecillinam group with positive vs negative urine culture.

Figure 9. Symptom score on day 0-7 for women in ibuprofen group with positive vs negative urine culture.
