# Supplementary figures and images for: Ibuprofen versus pivmecillinam for uncomplicated urinary tract infection in women—A double-blind, randomized non-inferiority trial
Source: PLoS Med. 2018 May 15;15(5):e1002569. doi: 10.1371/journal.pmed.1002569 (PMC5953442; doi:10.1371/journal.pmed.1002569)

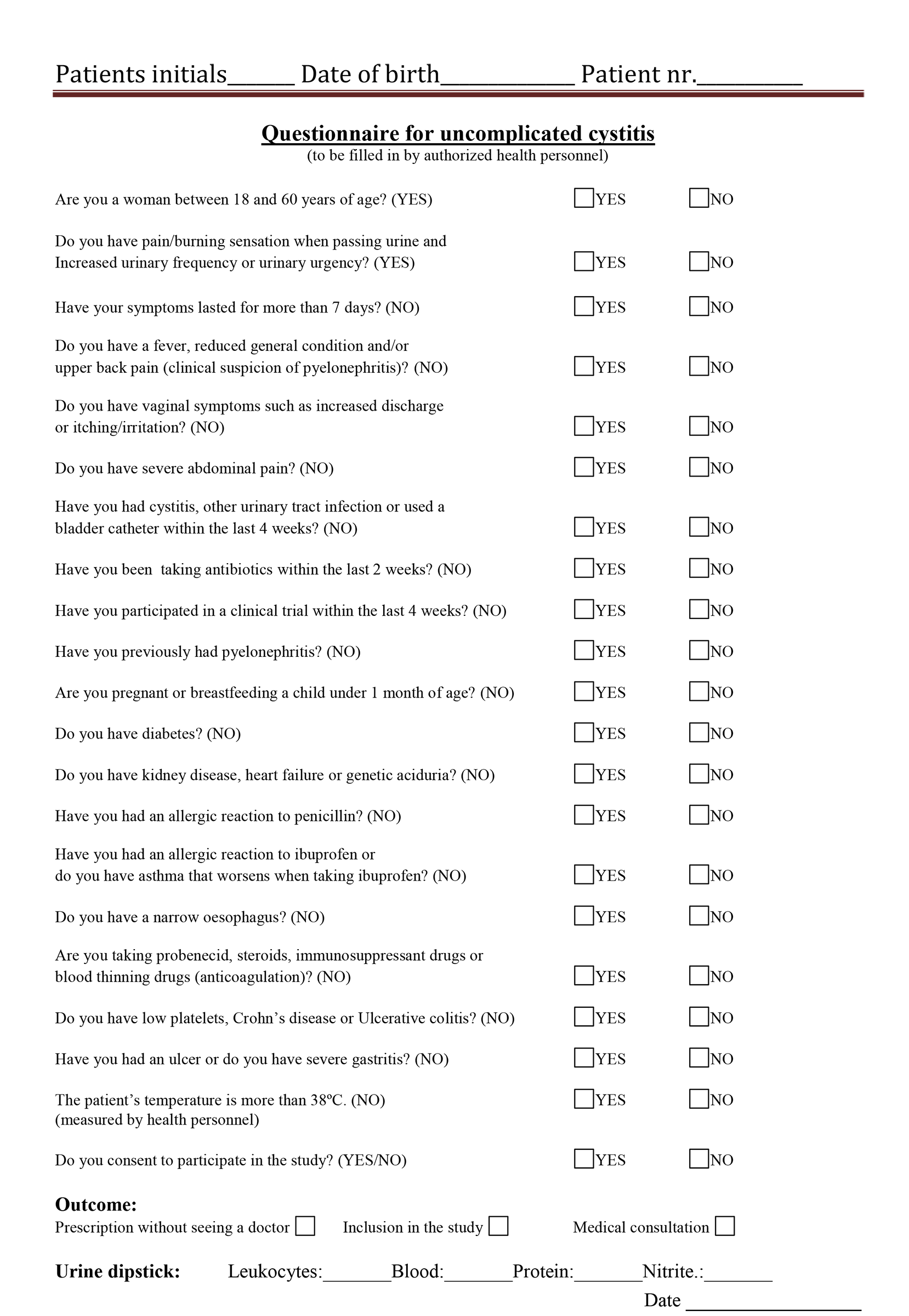

Supplement: S1 Fig — (TIF) [file pmed.1002569.s004.tif]

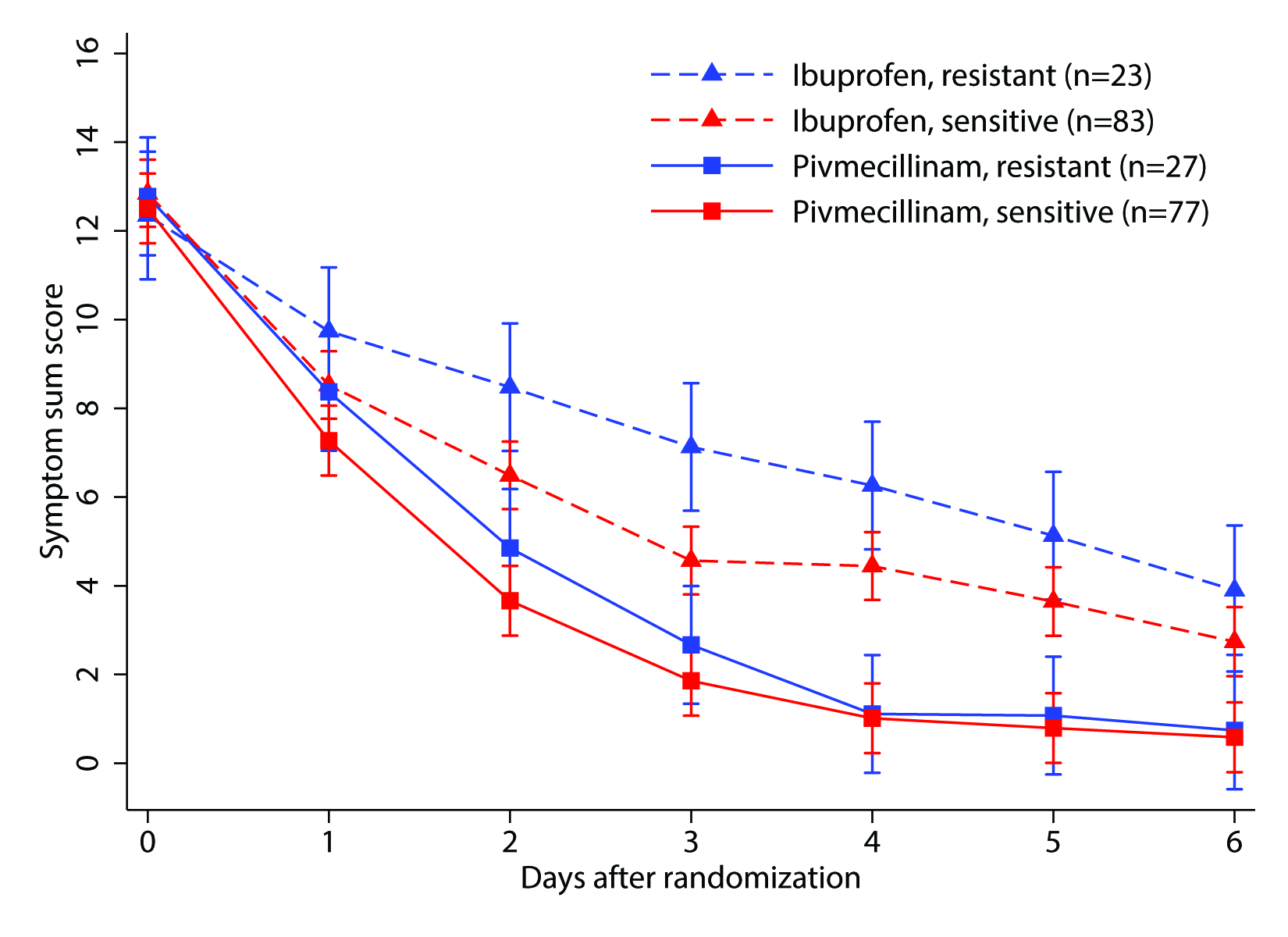

Supplement: S2 Fig — (TIF) [file pmed.1002569.s005.tif]

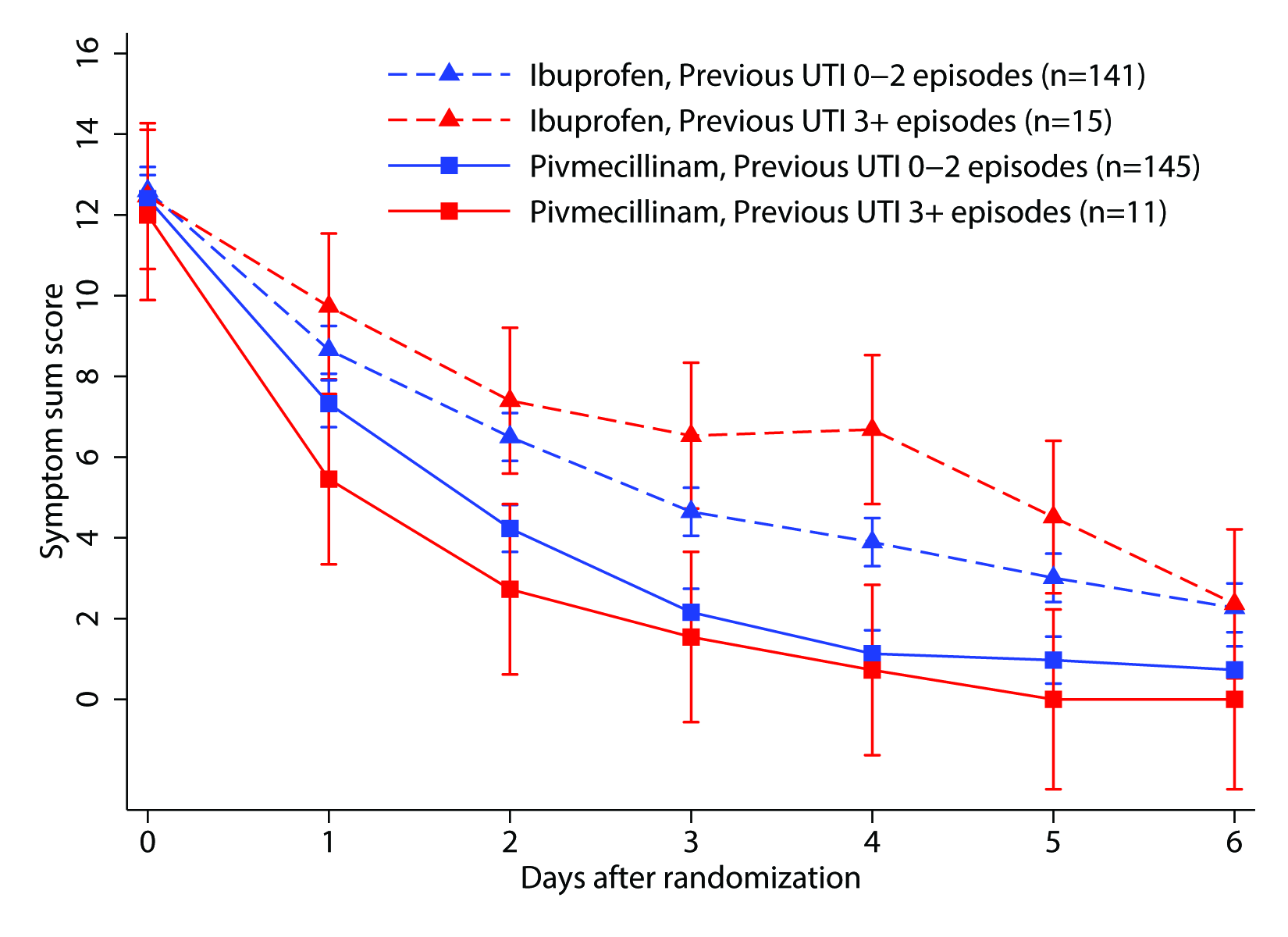

Supplement: S3 Fig — (TIF) [file pmed.1002569.s006.tif]

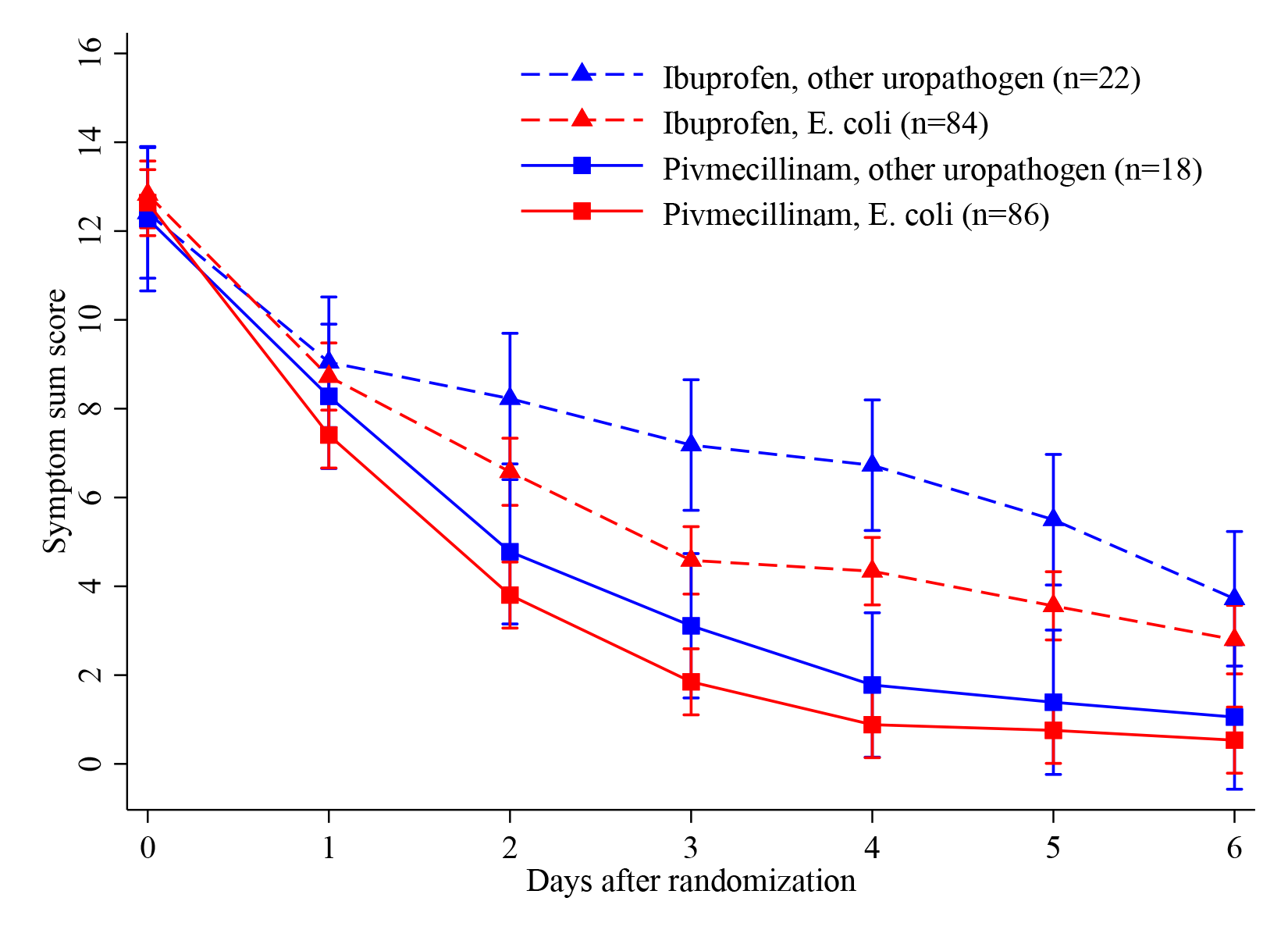

Supplement: S4 Fig — (TIF) [file pmed.1002569.s007.tif]
