# Supplementary material for: Ibuprofen versus pivmecillinam for uncomplicated urinary tract infection in women—A double-blind, randomized non-inferiority trial
Source: PLoS Med. 2018 May 15;15(5):e1002569. doi: 10.1371/journal.pmed.1002569 (PMC5953442; doi:10.1371/journal.pmed.1002569)
Supplement: S1 Protocol — (PDF) [file pmed.1002569.s008.pdf]

	Protocol, 4th edition, 19.03.2013<br><br>Ibuprofen versus mecillinam for uncomplicated cystitis in adult, non-pregnant women<br><br>A randomized controlled trial<br><br>EudraCTnr: 2012-002776-14

Sponsor
University of Oslo, Faculty of medicine, Institute of Health and Society

National coordinating investigator
Professor Morten Lindbæk, Faculty of medicine, Institute of Health and Society, Department of General Practice and Community Medicine, Antibiotic Centre for Primary Care, University of Oslo, Norway.

Principal investigator, Oslo, Norway
Professor Morten Lindbæk, Faculty of medicine, Institute of Health and Society, Department of General Practice and Community Medicine, Antibiotic Centre for Primary Care, University of Oslo, Norway.

Principal investigator, Bergen, Norway
Professor Anders Bærheim, Department of Public Health and Primary Health Care, University of Bergen, Norway.

Principal investigator, Lund, Sweden
Professor Sigvard Mölstad, Department of Clinical Sciences, Malmoe, General Practice, University of Lund, Sweden.

Principal investigator, Copenhagen, Denmark
Professor Lars Bjerrum, Department of public health, University of Copenhagen, Denmark,

Investigator and study coordinator
MD Ingvild Vik, Emergency Medical Agency of Oslo and Department of General Practice and Community Medicine, Antibiotic Centre for Primary Care, University of Oslo, Norway.

Monitor
A Monitor has not yet been appointed. We will collaborate with the centre for Good Clinical Practice at Oslo University Hospital in finding a Monitor and developing a monitoring plan for this study.

1. Relevance of the research project

Uncomplicated cystitis comprises 3-4% of all consultations in general practice. (1) The condition leads to more than 400.000 prescriptions of antibiotics every year. (The Norwegian institute of Public Health, Irene Litleskare, personal correspondence.)
The aim of this study is to compare an NSAID and an antibiotic in the treatment of uncomplicated cystitis in women. If we find that an NSAID is equally effective as an antibiotic, this may contribute to a reduction in the use of antibiotics and reduce antibiotic resistance globally. This is beneficial to the environment and will reduce costs in health services internationally.

2. Aspects relating to the research project

2.1. Background and status of knowledge

Uncomplicated cystitis is a superficial bacterial infection of the urinal bladder mucosa. It is a common infection in general practice, and much more common in women than in men, 100:1 in the age 20-60. (1) Research has been done, both nationally and internationally, that has mapped incidence, various treatments and development of bacterial resistance related to this condition. In general practice most women who present with symptoms of cystitis will fulfil the criteria for uncomplicated cystitis, and this is the most common bacterial infection in women. (2)

Research has shown that if a patient experiences dysuria and pollakiuria and/or urinary urgency and/or macroscopic hematuria and the symptoms have lasted less than seven days, it is very likely that she has cystitis. If she furthermore has no vaginal symptoms such as discharge or irritation, the likelihood increases to 90%. (3) Hence it is found that uncomplicated cystitis can be diagnosed and treatment can be prescribed over the telephone. (4) Twenty percent of all women report one or more episodes with dysuria during one year and 50% of all women will go through an uncomplicated cystitis during life. (5) Of all these women 20% will experience recurring infections. (6)

Although uncomplicated cystitis is considered to be a mild condition and often is self limiting, most patients who see a doctor will be treated with antibiotics. We know that most women with uncomplicated cystitis will be cured without any treatment, but antibiotics are known to give a quick relief of symptoms and shorten the course of the condition by a few days. The documentation for such an effect is, however, scarce. (7)

In Norway the national guidelines for treatment of uncomplicated cystitis recommend a three day treatment with an antibiotic. The antibiotic is chosen according to the National guidelines for antibiotic use in primary care, and today there are three kinds of antibiotics that are listed as equally good first choices, mecillinam, nitrofurantoin and trimethoprim. (8)
Previous epidemiological investigations of women with symptoms of cystitis in a general practice population have found *E. coli* to be the dominant bacterial agent (70-80%) and secondly *S. saprophyticus* (10-15%). (9-13)

A Norwegian retrospective study done on urine cultures from patients with urinary tract infection showed a statistically significant decrease in number of isolates of *E. coli* sensitive to antibiotics. The periods of comparison were 1997 to 1999 and 2003 to 2004. For hospitalized patients there was a decrease in sensitivity from 96% to 94% for mecillinam, from 97% to 95% for nitrofurantoin and from 73% to 71% for sulfonamide. For outpatients

the decrease in sensitivity was from 78% to 76% for ampicillin and from 83% to 82% for trimethoprim. The increase in resistant bacterial cultures can be seen as a result of an increased use of antibiotics. The study concluded that mecillinam and nitrofurantoin should be considered as first choices in treating uncomplicated cystitis because *E. coli* is the dominant agent and is most sensitive to these two substances. (14)

Several clinical trials have been performed to see whether the duration of antibiotic therapy in uncomplicated cystitis has any influence on the duration of the patients' symptoms. A systematic review of many of these trials has showed that three days of antibiotic therapy is as good as five to ten days in achieving symptomatic cure in these patients. The longer treatment is however more effective in obtaining bacteriological cure. Longer therapy is related to a higher rate of adverse reactions to the antibiotic used. (15)

A few trials have been made comparing the treatment of uncomplicated cystitis with antibiotics versus placebo. The results showed that the symptomatic improvement and bacteriological cure were significantly delayed in the placebo group, but at the same time the placebo group showed a symptomatic improvement rate of around 50% after three days. In these trials it seems that most uncomplicated cystitis is self limiting and that the patients become symptom free within a week. (16, 17)

A pilot study comparing the treatment of uncomplicated cystitis with ciprofloxacin to ibuprofen was recently published. (18) The study included 69 patients and it showed that 58% of the patients who received ibuprofen and 52% of the patients who received ciprofloxacin were asymptomatic after four days. Urine cultures taken on day seven showed more negative cultures among the patients who had received ciprofloxacin (72% vs. 49%), but the difference was not statistically significant.

The problem of rising bacterial resistance.
Most women who see a physician when they have symptoms of an uncomplicated cystitis will be given antibiotic treatment. This contributes to an increase in bacterial resistance, both in a population and individually. (14) This resistance in turn forces physicians to prescribe more broad spectrum antibiotics, such as ciprofloxacin. In the US and southern Europe ciprofloxacin has been used as a first choice for treating many infections, including uncomplicated cystitis. This has led to an increased rate of resistant strains, which means that physicians must prescribe even more broad spectrum antibiotics to treat simple infections. (19, 20) This is a problem not only for the patients as we run out of treatment options, but also for the environment as the global outlet of antibiotics into the nature is ever increasing and may alter the natural bacterial floras.

In a national and global perspective it is important to reduce the use of antibiotics. This project will hopefully show that in uncomplicated cystitis in adult, non-pregnant women, symptomatic treatment with an NSAID is equally effective as antibiotic treatment. If this is the case, antibiotic treatment may not be necessary for this uncomplicated infection. As uncomplicated cystitis is a very common condition among women world wide, this will be an important contribution to help reducing the use of antibiotics on a global scale.

2.2. Approaches, hypotheses and choice of method

The aim of this study is to evaluate ibuprofen versus mecillinam in the treatment of uncomplicated cystitis in otherwise healthy, adult, non-pregnant women.

Our hypothesis is that symptomatic treatment with NSAIDs is not inferior to antibiotic treatment in this group. Another unanswered question is whether NSAIDs (ibuprofen) might have an antimicrobial effect.

To our knowledge no large randomized controlled trial (RCT) has previously been done to explore this. There was a pilot study in Germany in 2010 (18), and the same group has just started recruiting patients to a new RCT in Germany where they will compare treatment with ibuprofen to fosfomycin-trometamol. Also a group in Switzerland has planned to do an RCT where they will compare treatment with diclofenac to norfloxacin, but they have not started recruiting patients yet. (21) Both these studies have chosen to use quite broad spectrum antibiotics. We have chosen mecillinam which is a narrow spectrum antibiotic, it has a relatively low resistance driving effect, beneficial adverse effect profile and it is approved for use in the first trimester of pregnancy in the National guidelines for antibiotic use in primary care. (8) We have chosen to use the NSAID ibuprofen over other NSAIDs because of its relatively beneficial adverse effect profile and the dosage we will be using is lower than the maximum recommended daily intake.

The design of the study will be a double blinded RCT. Half of the patients will receive treatment with mecillinam and the other half will receive treatment with ibuprofen. The study will follow the principles of Good Clinical Practice (GCP).

Urine cultures will be obtained on day one and after two weeks. If we demonstrate that the proportion of patients with persistent bacteriuria is equal in the two groups, this will show that the immune system of the women in the ibuprofen group may have handled the bacteria without antibiotics, or that ibuprofen may have an antimicrobial effect.

The patients will be given a diary where they daily will register symptom load, possible complications or adverse effects and on which day they feel completely cured. We will contact the patients after two weeks to make sure they have followed the study procedures. After four weeks we will perform a final interview with the patient.

<u>Main outcome measures</u>

Proportion of patients who felt cured on day four as registered in the patient diary.

<u>Secondary outcome measures</u>

The patients' symptom load with regard to specific symptoms.
Proportion of patients who were in need of a secondary medical consult within the study period.
Proportion of patients who developed an upper urinary tract infection (pyelonefritis).
Proportion of patients who experienced adverse effects.
Proportion of patients who experienced severe adverse effects.
Proportion of patients who had a relapse of symptoms within four weeks after being included in the study.
Proportion of patients with a positive urine culture after two weeks.

The measurements in this study will be based on information from the patient's diary, the result of the urine cultures taken on day one and after two weeks and telephonic contact with the patient after two and four weeks. We will register whether the patient felt cured after the

study treatment or if she needed a second medical consultation. If she had a second medical consultation we will register what kind of treatment she received. We will also register whether the patient has had a relapse of symptoms of an uncomplicated cystitis within four weeks after joining the study. We will also register whether the patient has taken any additional NSAIDs during the first two weeks after inclusion. If she has, this might influence the study results with regards to finding whether ibuprofen can have an antibiotic effect and thus lead to exclusion from the study.

From the urine cultures we can measure how many of the patients who feel cured on day four actually have negative urine cultures and how many have asymptomatic bacteriuria. And if they have asymptomatic bacteriuria, are they more likely to get a new episode of cystitis? We would also like to see how many of the patients who still have symptoms after day four actually have positive urine cultures. And if they do, are they more likely to develop an upper urinary tract infection (UTI) or will they continue having symptoms of an uncomplicated cystitis and just need a few days longer to feel cured.

Does ibuprofen have an antimicrobial effect?
As a sub study it would be of great interest to investigate whether ibuprofen has an actual antimicrobial effect. To do this, it will first be necessary to develop a new test series in the bacteriological laboratory. It is necessary to test breaking points for the antimicrobial effect of ibuprofen in urine samples contaminated with some of the most common urine bacteria such as *E. coli*, *Klebsiella*, *S. saprophyticus* and *Enterococci*.
If we find such an effect we would like to go further and also use the urine cultures to investigate whether ibuprofen could have an antimicrobial/antibiotic effect. In order to do so we will collaborate with the microbiological laboratory at Oslo University Hospital, Ullevål and possibly also at Vestfold Hospital and laboratories in Sweden and Denmark. The details of such a study will be described in a particular protocol at a later stage.

Sample size
We assume that 85% in the mecillinam group will feel completely cured after four days and consider a 10% absolute reduction to 75% in the ibuprofen group as a maximum relevant difference when holding that ibuprofen is non inferior to mecillinam. With alpha 0,05 and 1-beta of 80% we estimate that we need about 150 patients in each group. To add up for drop outs and withdrawals we aim at recruiting 200 in each group. Stratification is not found to be necessary.
85% is based on an ongoing study on uncomplicated cystitis at the Emergency Medical Agency (EMA) in Oslo where 85% of patients felt cured after four days. (Marianne Bollestad, personal communication)

2.3. The project plan, project management, organisation and cooperation

Study organization and cooperation
The research project is a co-work between Department of General Practice and Community Medicine and Antibiotic Centre for Primary Care, University of Oslo, Norway, the Department of Public Health and Primary Health Care, University of Bergen, Norway, the Department of public health, Copenhagen University, Denmark, and the Department of Clinical Sciences, Malmoe, General Practice, Lund University, Sweden.

The study is an RCT with two arms and double blinding. The study will be recruiting patients from the EMAs in Oslo and Bergen, Norway, some general practices in Denmark, and some general practices in Sweden.

The four research networks all have a strong infrastructure. All the four principal investigators have long experience in performing RCTs in a primary care setting, some of them being large and recruiting many patients over a long period of time. Some of these studies have been performed through international networks and have managed to handle all the challenges this has entailed.

Setting and participants

To recruit patients to this study the patients who present with symptoms of an uncomplicated cystitis will be given a questionnaire to see whether they qualify for being included in the study. The questionnaire will be answered by the patient in front of a trained research nurse who can answer any questions they might have. This questionnaire/form is already in use at the EMA in Oslo and we intend to use the same questionnaire/form at all inclusion sites. The patients who qualify for being included in the study will be given information about the study, both orally and in writing. If they decide to participate, they will be given a consent form which will be signed both by the patient and the research nurse. The consent form is made in accordance with the guidelines of the Regional Ethical Committee in Norway (REK). We aim at recruiting 200 patients at the EMA in Oslo, and 70 patients at each of the three other sites. We hope to finish recruiting patients through 2015. A pilot study will be performed at the EMA in Oslo in March - April 2013 to test logistics and all the forms. We aim at including 30 patients in the pilot study. If the pilot study does not imply major changes in the protocol, these patients will be included in the main study in addition to the 200 we aim to recruit in the main study.

Inclusion criteria:

- woman between 18 and 60 years of age
- dysuria and pollakiuria and/or urinary urgency
- ability to give written consent

Exclusion criteria:

- pregnancy/breastfeeding child under one month of age
- diabetes
- kidney disease
- genetic aciduria
- clinical suspicion of pyelonefritis; fever, reduced general condition, upper back pain
- vaginal symptoms such as discharge or irritation
- severe abdominal pain
- symptoms that have lasted for more than seven days
- one or more urinary tract infections within the lasts four weeks
- permanent bladder catheter or use of bladder catheter within the last four weeks
- use of antibiotics within the last two weeks
- participated in a clinical trial within the last four weeks
- previously undergone a pyelonefritis
- previous allergic reaction to penicillin
- previous allergic reaction to ibuprofen, or worsening of asthma when using NSAIDs
- narrow oesophagus

- use of the drug probenecid
- severe gastritis or previous ulcer
- anticoagulative treatment
- ongoing use of steroids
- use of immunosuppressant drugs
- thrombocytopenia
- heart insufficiency
- severe psychiatric illness or dementia
- severe drug addiction
- unable to communicate in Norwegian, Swedish or Danish language

The last three exclusion criteria will not be included in the questionnaire, but the study nurse will evaluate this during the consultation with the patient.

Other than the drugs listed in the exclusion criteria there are no known significant drug interactions for the study medicine in this study. The patient shall continue taking all prescription drugs as usual throughout the trial. They will be asked to register all prescription drugs in the patient diary. There will be no restrictions as to whether the patient can take non prescription medicine or nutritional supplements during the study, but if they are taking any they are asked to register this in the patient diary.

Blinding and randomization
We want to make the study double blinded. We will buy the original tablets directly from the manufacturers in the pharmaceutical industry, Ibuprofen ratiopharm (ibuprofen) 600 mg from Ratiopharm and Selexid (mecillinam) 200 mg from Leo Pharma. Kragerø Tablettproduksjon AS, a company in Kragerø, Norway, will over-encapsulate the medicine to be used in this study. The original tablets will be put in a gelatine capsule. The capsules will then be filled with small balls of sugar so that the capsules become as similar as possible. The capsule consists of two parts which will be put together and firmly tightened to make it as difficult as possible to open. We have already looked at the final product and this procedure will give the capsules the same look, feel, weight and taste.

The study medicine will be packed in two different kits, one with 9 capsules containing one tablet of mecillinam each, the other with 9 capsules containing one tablet of ibuprofen each. Each kit will then be labelled with a study number. This labelling will be done following a randomization list made in advance using block randomization. Kragerø Tablettproduksjon AS will send all documentation regarding the study medicine to the Norwegian Medicines Agency (NoMA) for approval.

At inclusion the patient receives a kit labelled only with the study number. Neither the research nurse nor the patient will know what active substance is given, thus resulting in double blinding for both nurse/doctor and patient. A list linking the study number to the active substance is kept at Kragerø Tablettproduksjon AS. The list will be retrieved only at the end of the study when all the data has been collected and entered into the statistical program and all analysis are completed.

Handling of the study medicine
The study medicine will be packed and labelled at Kragerø Tablettproduksjon AS. They will send the study medicine to each of the study sites according to the randomization list. At each

study site the study medicine will be handled according to existing local routines and guidelines. The study medicine will be kept in a locked facility.

Intervention

Day 1

The research nurse fills out the questionnaire together with the patient. The questionnaire will give us information about symptom load at inclusion. The questionnaire is an appendix to the protocol.

The patient and research nurse sign the consent form. The consent form is an appendix to the protocol.

The patients are randomized into two groups. Both groups receive active treatment and none are given placebo. One group will be given mecillinam 200 mg 3 TID for three days and the other will be given ibuprofen 600 mg 3 TID for three days.

The research nurse will take a urine dipstick (leukocytes, nitrite and blood) and register the result in the patient journal. A urine culture will be sent to the laboratory and the patients will receive equipment to take a new urine sample at home and deliver it at the study inclusion site for a second culture after two weeks.

Day 1-7

The patients receive a diary where they are going to register the severity of symptoms on a daily basis and on which day they feel completely cured. They will also register possible adverse effects or complications and whether they finished the three day treatment with study medicine or not. If they didn't finish the treatment we will ask how many capsules they have left. When the patients have completed the diary they will send it to or delivered it at the study site. The patient diary is an appendix to the protocol.

Day 14

The patient will deliver a second urine sample for a control urine culture. A research nurse will call the patients after two weeks for a follow up interview. If they have not yet sent us the diary or delivered a second urine sample, they will be reminded to do this.

Day 28

A research nurse will perform a short telephonic interview with questions about possible relapse of symptoms and need for additional treatment.

The patients will be instructed only to use paracetamol if they have the need for additional pain relief. They are not allowed to take any NSAIDs during the study period as this can interfere with our measurements. If we find they have been taking additional NSAIDs during the study period this will lead to exclusion from the study.

The patients will be asked to register all their prescription drugs in the patient diary. They are also asked to register whether they are taking any nutritional supplements or non prescription drugs.

Criteria for termination of the study

For the individual patient

- developing an upper UTI in need of hospitalization
- serious adverse effect or allergic reaction to the study medicine
- a wish to withdraw from the study

For the study itself

- when the sufficient number of patients have been included
- if more than ten patients within the first 100 patients included are in need of hospitalization, this will be a cause for unblinding of these patients. This is to see whether there are significantly more patients in the ibuprofen group who were in need of hospitalization, and if so we will have to consider terminating the study

Second medical consult

If a patient experiences getting worse or not getting better, she is instructed to contact either the study doctor for advice or the study site for a second medical consult. Contact information and routines are described in the patient information in the consent form. There will also be written routines for the health personnel at each study site to follow when a study patients comes in for a second medical consult.

If a patient contacts us having decided to quit the study because she wants to be sure she is given antibiotic treatment, she will be given mecillinam for three days. These patients will be included in the study according to the principle of intention to treat (ITT).

If a patient experiences continuing symptoms of an uncomplicated cystitis (more than seven days), but no signs of developing an upper UTI, she will be offered a second treatment, this time with another antibiotic treatment. The patient will be given either trimethoprim or nitrofurantoin for three days. The treatment is chosen according to resistance pattern in the urine culture from day one and/or eventual allergies. These patients will stay included in the study according to the principle of ITT.

If a patient shows signs of developing an upper UTI, but is in good general condition, she will be offered an antibiotic treatment with either trimethoprim-sulpha or ciprofloxacin for ten days. Here too the treatment is chosen according to resistance pattern in the urine culture from day one and/or eventual allergies. These patients will also stay included in the study according to the principle of ITT.

If a patient develops a serious upper UTI and is in need of hospitalization, the doctor treating the patient will make it clear to the hospital that she is participating in a clinical trial. If the resistance pattern in the urine culture is already known this information will be given to the hospital. If the doctor at the hospital needs to know what active treatment the patient has received, this information will be provided. These patients will stay included in the study according to the principle of ITT.

Adverse effects.

We expect some gastrointestinal adverse effects for both treatments. All adverse effects will be registered in the patient diary. When registering the data from the study the adverse effects will be divided into three groups, adverse events (AE), serious adverse events (SAE) and suspected unexpected serious adverse reaction (SUSAR). If the patient should experience an allergic reaction or a severe adverse effect, she is instructed to contact the study centre or the local emergency unit immediately. These conditions demand a change of active treatment and may be a cause to reveal the active substance this patient has been given.

A box containing an envelope for each study number will be kept at each study centre. Each sealed envelope, marked only with the study number, contains a note with the name of the active substance given to the patient with this study number. If a patient experiences an

allergic reaction or a severe adverse event that demand knowledge of active treatment, the envelope for this study number will be opened and the patient and the patient and the doctor treating the patient will be informed of what active substance the patient has received. The doctor responsible for the study will not be informed. The information will be revealed to a research nurse. She will report the event to the doctor responsible for the study without revealing the study number or the active substance given. The doctor will decide if the event is classified as a SAE or a SUSAR. If it is classified as a SUSAR a designated person working for the Sponsor will be contacted by the study nurse and will be given all the information about the patients' study number, the active substance given and the event itself. This designated person will inform the Sponsor and then file a report about the SUSAR to the authorities. The report will be filed within seven to 15 days after the Sponsor has been made aware of the event, depending on the severity of the SUSAR. This is in accordance with Regulations on clinical trials of pharmaceuticals in humans. (24)

All AE, SAE and SUSAR will be reported from each study site to the Sponsor. The Sponsor will report all SAE and SUSAR in an annual report to the authorities using report forms standardized by the Oslo University Hospital according to the principles of GCP and in accordance with Regulations on clinical trials of pharmaceuticals in humans. (24) When the study is finished the Sponsor will file a final report to the authorities.

Registering and handling of data
An investigators study file (ISF) will be established at each of the study sites. A trial master file (TMF) is established in Oslo by the national coordinating investigator.
The ISF will contain all the essential information about the study.

The registering of data will be made consecutively throughout the study period. The source data will be registered in a case report form (CRF) for each patient according to the principles of GCP. All the documents in the CRF will be marked with the study number and the patients' initials. Only the consent form will also contain the patient's name and date of birth. The CRF itself will be marked only with the study number. The CRF's will be kept in a locked facility with limited access. The handling of the data will follow the principles of GCP. The data will be kept in a locked facility for 15 years after the study is finished. After this it will be destroyed.

Source data / source documents
Questionnaire
Consent form
Urine dipstick day 1
Urine cultures day 1 and day 14
Patient diary
Telephonic contact day 14 and 28

Statistical analyses
Data will be registered and analyzed in statistics program SPSS version 16. We will do descriptive analysis and logistic regression analysis of main and secondary outcome measures. Patients who experience an allergic reaction or a SAE or a SUSAR will be included in the study according to the principle of ITT. Patients who take additional NSAIDs or who whish to withdraw from the study without handing in the patient diary or a second urine sample will be excluded from the study.

The main form of analyzing the results will be ITT, but per protocol analysis will also be made. The analysis will be performed in cooperation with a statistician at the University of Oslo.

Monitoring
This study will be carried out according to the principles of GCP, the latest revision of the Declaration of Helsinki and national laws and regulations.

The monitor will perform the first visit within a month after at least 10 patients have been included at each study site. After this the monitoring visits will be performed as frequently as needed following communication between the principal investigators and the monitor. At every visit the monitor will check all the essential documents in the ISF, the signed consent forms and make sure the different sections in the CRF are correctly filled out.

If necessary the investigators and the monitor will seek advice from Section for GCP at Oslo University Hospital.

3. Key perspectives and compliance with strategic documents

3.1. Compliance with strategic documents

The Norwegian national strategic plan for fighting antibiotic resistance has asked for more studies done in the primary care setting, studying the most common bacterial illnesses. Our study is in line with this. (22, 23)

3.2. Relevance and benefit to society

As uncomplicated cystitis is the most common bacterial infection in adult women, this represents a large portion of the consultations at a general practice. If we could reduce the need for antibiotic treatment for this condition, and maybe find that it can be treated with a simple over-the-counter drug such as ibuprofen, this will reduce the number of consultations for this condition and hence reduce the total costs both for the patient and the national health service. It will also contribute to a reduction in the development of antibiotic resistance, both in the community and in the patient herself.

3.3. Environmental impact

The global outlet of antibiotics into nature is ever increasing and interfering with the natural bacterial floras. In a national and global perspective it is important to reduce the use of antibiotics. This project will hopefully show that in uncomplicated cystitis in adult, non-pregnant women, symptomatic treatment with a NSAID is equally efficient as antibiotic treatment. Thus antibiotic treatment may not be necessary in this uncomplicated infection. As uncomplicated cystitis is a very common condition among women world wide this will be an important contribution to help reduce the use of antibiotics in a global perspective.

3.4. Ethical perspectives

Uncomplicated cystitis is predominantly a self limiting condition and the need for antibiotic treatment of this condition is controversial. However the condition is often painful and the patients feel the need to take pain medication, often NSAIDs.
In this study half of the patients will receive an antibiotic (mecillinam) and the other half will receive an NSAID (ibuprofen), they will also have the opportunity to take paracetamol in addition to the study medicine, so no one should suffer unnecessary pain.

The risk of developing a more serious infection despite not receiving antibiotic treatment is considered to be small. The pilot study in Germany showed that 33,3% of the patients in the NSAID group and 18,2% in the antibiotic group required secondary antibiotic treatment. The difference was notable, but not statistically significant. None of the patients were in need of hospitalization. No patients experienced severe adverse effects. (18)

If the patient qualifies for inclusion in the study she will receive information both orally and in writing. It will be pointed out that participation is completely voluntary and that declining does not affect the kind of help or treatment she will receive. It will be made clear to the patient that she can choose to quit the study at any time. If the patient has any questions regarding the study she can contact a research nurse or a research doctor at the study site. The phone numbers are listed in the consent form received and signed at inclusion.

It will be emphasized that if the patient gets worse during the study treatment or experiences any severe adverse effects, she must contact the study site or come back for a medical consultation. Preferably at the site of inclusion, otherwise at the local emergency unit or other institution with a special agreement with the local study site. Information about such institutions will be made very clear, both orally and in the consent form.

Given this we believe that the patients in this study will be well taken care of and it is ethically justifiable to carry out this study.

The protocol will be sent to the Regional Ethical Committee in Norway (REK) and the Norwegian Medicines Agency (NoMA) for approval. The study will be registered in clinicaltrials.gov.

3.5. Gender issues (Recruitment of women, gender balance and gender perspectives)
Uncomplicated cystitis is a common reason for women all around the world to see a physician. If we can show that this condition is easily treated with a simple over-the-counter drug such as ibuprofen and that antibiotics are not needed, this will benefit women’s health on a global scale.

4. Dissemination and communication of results

4.1 Dissemination plan
The results will be published in international journals and at international congresses. It will also be communicated to Public health authorities.

4.2 Communication with users
In addition to publication of scientific articles we will contact the public health authorities and use the mass media to convey our results.

5. Economy and insurance
The project will be financed through different research funds. So far we have applied to the National Centre for Emergency Primary Health Care and the Norwegian Research Council.

A collective insurance will be taken out through the Norwegian drug liability insurance for all participants in the study. The participants are also insured/covered through the national health service system.

6. Confirmation

We hereby confirm that the study will be carried out according to the protocol, Good Clinical Practice (GCP) and according to national guidelines, laws and regulations.

Oslo, 19.03.13

Best regards

Morten Lindbæk
Professor
National coordinating investigator

Ingvild Vik
MD
Investigator and study coordinator

Anders Bærheim
Professor
Principal investigator, Bergen, Norway
Date:

Sigvard Mölstad
Professor
Principal investigator, Lund, Sweden
Date:

Lars Bjerrum
Professor
Principal investigator, Copenhagen, Denmark
Date:

7. References

1. Allmennmedisin (Textbook of general practice), Hunskår S (ed.), 2003, p566
2. Colgan R, Williams M. Diagnosis and treatment of acute uncomplicated cystitis. Am. Fam. physician 2012, 84: 771-6.
3. Bent S, Nallamothu BK, Simel DL, et al. Does this woman have an acute uncomplicated urinary tract infection? JAMA 2002; 287: 2701-10.
4. Barry HC, Hickner J, Ebell MH, Ettenhofer T. A randomized controlled trial of telephone management of suspected urinary tract infections in women. J Fam Pract 2001;50:589-594.
5. Nicolle LE. Epidemiology of urinary tract infection. Infect. Med. 200, 18: 153-62
6. Stamm WE, McKevitt M, Roberts PL, White NJ.Natural history of recurrent urinary tract infections in women. Rev. Infect. Dis. 1991; 13: 77-84.
7. Flottorp S, Oxman AD, Cooper JG et al. Retningslinjer for diagnostikk og behandling av akutte vannlatingsplager hos kvinner (Guidelines for diagnosing and treating acute cystitis in women). Tidsskr Nor Legeforening 2000, 120: 1748-53
8. Nasjonale faglige retningslinjer for antibiotikabruk i primærhelsetjenesten (National guidelines for antibiotic use in primary care). Lindbæk M, red. Oslo, Helsedirektoratet 2008.
9. Agdestein B, Lindbæk M, Gjelstad S.Følges retningslinjene ved antibiotikaforskrivning mot urinveisinfeksjon? (Are guidelines for antibiotic treatment for urinary tract infection followed?) Tidsskrift for Den norske legeforening nr 17, 2011.
10.NORM/NORM-VET 2010, Usage of antimicrobial agents and occurrence of antimicrobial resistance in Norway. Tromsø/Oslo NORM/NORM-VET 2010.
11. Grude N, Tveten Y, Jenkins A et al, Uncomplicated urinary tract infections. Bacterial findings and efficacy of empirical antibacterial treatment. Scan J Prim Health Care 2005, 23: 115-9.
12. Jureen R, Digranes A, Baerheim A, Urinveispatogene bakterier ved ukomplisert nedre urinveisinfeksjon hos kvinner (Urinary tract pathogens in uncomplicated lower urinary tract infections in women). Tidsskr Nor Legeforening 2003, 123: 2012-2.
13. Grude N, Tveten Y, Kristiansen B-E, Urinary tract infections in Norway: bacterial etiology and susceptibility. A retrospective study of clinical isolates. Clin Microbiol Infect 2001; 7: 543-7
14. Skudal H, Grude N, Kristiansen B-E. Økende forekomst av antibiotikaresistens ved urinveisinfeksjoner (Increasing prevalence of antibiotic resistance in urinary tract infections) . Tidsskrift for Den norske legeforening nr.8, 2006.
15. Milo G, Katchman EA, Paul M, Christiaens T, Baerheim A, Leibovici L.Duration of antibacterial treatment for uncomplicated urinary tract infection in women (Review). The Cochrane Collaboration. The Cochrane Library 2005, issue 2.
16. Christiens TC, De Meyere M, Verschraegen G et. al. Randomized controlled trial of nitrofurantoin versus placebo in the treatment of uncomplicated urinary tract infection in adult women.
17. Ferry SA, Holm SE, Stenlund H, Lundholm R, Monsen TJ. The natural course of uncomplicated lower urinary tract infection in women illustrated by a randomized placebo controlled study. Scand. Infect. Dis. 2004, 36: 296-301.
18. Bleidorn et al. Symptomatic treatment (ibuprofen) or antibiotics (ciprofloxacin) for uncomplicated urinary tract infection? Results of a randomized controlled pilot trial. BMC medicine 2010.
19. Siddiqui AA. Prevalence of quinolone-resistant urinary tract infections in Comanche County hospital. J Okla State Med Assoc. 2008, Sep, 101(9):1150-8.
20. Mc Quilkin M, Lund A, Palmer W.Antimicrobial resistance of uncomplicated urinary tract infections in northern Utah. Clin Lab Sci. 2008 Spring; 21(2):99-101.
21. www.clinicaltrials.gov
22. Ny strategi for forebygging av infeksjoner i helsetjenesten og antibiotikaresistens (New strategy for prevention of infections in the health service and antibiotic resistance) (2008-2012)
23. St.Meld. St.(2011-2012), Global helse i utenriks- og utviklingspolitikken (Global health in foreign and development politics). Tilrådning fra Utenriksdepartementet 3. februar 2012, godkjent i statsråd samme dag. (Regjeringen Stoltenberg II)
24. Forskrift til klinisk utprøving av legemidler til mennesker. www.lovdata.no/cgi-wift/ldles?doc=/sf/sf/sf-20091030-1321.html
