# Supplementary material for: Ibuprofen versus pivmecillinam for uncomplicated urinary tract infection in women—A double-blind, randomized non-inferiority trial
Source: PLoS Med. 2018 May 15;15(5):e1002569. doi: 10.1371/journal.pmed.1002569 (PMC5953442; doi:10.1371/journal.pmed.1002569)
Supplement: S1 Table — Figures are number of women (percentage) unless stated otherwise. (DOCX) [file pmed.1002569.s009.docx]

S1 Table. Summary of primary and key secondary outcomes in women with uncomplicated UTI randomized to either ibuprofen or pivmecillinam, per protocol population. Figures are number of women (percentage) unless stated otherwise

| Outcomes | Ibuprofen (n=150) | Pivmecillinam (n=154) | Adjusted risk difference (95% CI) |
| --- | --- | --- | --- |
| Primary outcome |  |  |  |
| Patients without symptoms by day 4 | 60 (40) | 112 (73) | 33% (22 to 43%) |
| Secondary outcomes |  |  |  |
| Patients without symptoms by day 7 | 96 (64) | 138 (90) | 26% (16 to 35%) |
| Patients without symptoms by day 14 | 120 (80) | 143 (93) | 13% (5 to 20%) |
| Median symptom duration after randomisation | 6 | 3 |  |
| *Urine cultures after 14 days** |  |  |  |
| Urine culture positive | 37 (27) | 15 (10) | -15% (-25 to -6%) |
| Growth of primary pathogens | 25 (18) | 5 (3) | -13% (-22 to -6%) |
| *Relapses/complications** |  |  |  |
| Treatment with antibiotics within day 14 | 56 (38) | 13 (9) | -28% (-37 to -19%) |
| Treatment with antibiotics within day 28 | 63 (43) | 16 (11) | -31% (-40 to -21%) |
| Patients with febrile UTI† | 4 (3) | 0 (0) | -2.7% (-6.7 to 0.3%) |
| Patients with pyelonephritis† | 4 (3) | 0 (0) | -2.7% (-6.7 to 0.3%) |
| Serious adverse events | 3 (2) | 1 (1) | -1.4% (-4.2 to 1.4%) |

*Numbers represent observed (non-missing) data while estimates are based on imputed data

† Complications categorized as no complications, febrile UTI and pyelonephritis. Treatment differences are presented with unadjusted confidence limits using Newcombe's hybrid score method.
