# Supplementary material for: Ibuprofen versus pivmecillinam for uncomplicated urinary tract infection in women—A double-blind, randomized non-inferiority trial
Source: PLoS Med. 2018 May 15;15(5):e1002569. doi: 10.1371/journal.pmed.1002569 (PMC5953442; doi:10.1371/journal.pmed.1002569)
Supplement: S2 Table — Figures are proportions (percentage) unless stated otherwise. (DOCX) [file pmed.1002569.s010.docx]

| Initial infecting organism | Ibuprofen | Pivmecillinam |
| --- | --- | --- |
| Negative/no significant growth | 19/59 (32.2%) | 3/64 (4.7%) |
| *E. coli* | 50/95 (52.6%) | 12/93 (12.9%) |
| *S. saprophyticus* | 9/17 (52.9%) | 3/12 (25.0%) |
| *Enterococcus faecalis* | 0/2 (0.0%) | 0/0 (%) |
| *Klebsiella pneumoniae* | 2/2 (100.0%) | 2/2 (100.0%) |
| *Proteus mirabilis* | 1/1 (100.0%) | 0/0 (%) |
| *Enterobacter species* | 1/2 (50.0%) | 0/4 (0.0%) |
| *Citrobacter koseri* | 2/2 (100.0%) | 0/0 (%) |
| Other uropathogens | 0/0 (%) | 0/2 (0.0%) |

S2 Table. Need for secondary antibiotic treatment by initial infecting organism, intention to treat population of women with uncomplicated UTI randomized to either ibuprofen or pivmecillinam. Figures are proportions (percentage) unless stated otherwise
