# Supplementary material for: Ibuprofen versus pivmecillinam for uncomplicated urinary tract infection in women—A double-blind, randomized non-inferiority trial
Source: PLoS Med. 2018 May 15;15(5):e1002569. doi: 10.1371/journal.pmed.1002569 (PMC5953442; doi:10.1371/journal.pmed.1002569)
Supplement: S3 Table — (DOCX) [file pmed.1002569.s011.docx]

S3 Table. Baseline urine culture (down) versus second urine culture (horizontal), intention to treat population of women with uncomplicated UTI randomized to either ibuprofen or pivmecillinam

| Group | First culture organism | Second culture organism | | | | | | | |
| --- | --- | --- | --- | --- | --- | --- | --- | --- | --- |
|  |  | Negative/no significant growth | *E. coli* | *S. saprophyticus* | *Enterococcus faecalis* | *Klebsiella pneumoniae* | *Proteus mirabilis* | Other uropathogens | Missing |
| Ibuprofen | Negative/no significant growth | 41 | 7 | 0 | 1 | 0 | 0 | 2 | 8 |
|  | *E. coli* | 56 | 20 | 0 | 2 | 0 | 0 | 4 | 13 |
|  | *S. saprophyticus* | 12 | 0 | 2 | 0 | 0 | 0 | 1 | 2 |
|  | *Enterococcus faecalis* | 0 | 0 | 0 | 0 | 0 | 0 | 0 | 2 |
|  | *Klebsiella pneumoniae* | 0 | 0 | 0 | 1 | 1 | 0 | 0 | 0 |
|  | *Proteus mirabilis* | 0 | 0 | 0 | 0 | 0 | 0 | 0 | 1 |
|  | *Enterobacter species* | 2 | 0 | 0 | 0 | 0 | 0 | 0 | 0 |
|  | *Citrobacter koseri* | 0 | 0 | 0 | 0 | 0 | 0 | 1 | 1 |
|  | Other uropathogens | 0 | 0 | 0 | 0 | 0 | 0 | 0 | 0 |
|  | Missing | 0 | 0 | 0 | 1 | 0 | 0 | 0 | 0 |
| Pivmecillinam | Negative/no significant growth | 51 | 2 | 0 | 0 | 0 | 0 | 3 | 8 |
|  | *E. coli* | 71 | 4 | 0 | 2 | 0 | 1 | 4 | 11 |
|  | *S. saprophyticus* | 10 | 0 | 0 | 0 | 0 | 0 | 0 | 2 |
|  | *Enterococcus faecalis* | 0 | 0 | 0 | 0 | 0 | 0 | 0 | 0 |
|  | *Klebsiella pneumoniae* | 2 | 0 | 0 | 0 | 0 | 0 | 0 | 0 |
|  | *Proteus mirabilis* | 0 | 0 | 0 | 0 | 0 | 0 | 0 | 0 |
|  | *Enterobacter species* | 2 | 0 | 0 | 0 | 0 | 0 | 0 | 2 |
|  | *Citrobacter koseri* | 0 | 0 | 0 | 0 | 0 | 0 | 0 | 0 |
|  | Other uropathogens | 1 | 0 | 0 | 0 | 0 | 0 | 0 | 1 |
|  | Missing | 1 | 0 | 0 | 0 | 0 | 0 | 0 | 0 |
