# Supplementary material for: Ibuprofen versus pivmecillinam for uncomplicated urinary tract infection in women—A double-blind, randomized non-inferiority trial
Source: PLoS Med. 2018 May 15;15(5):e1002569. doi: 10.1371/journal.pmed.1002569 (PMC5953442; doi:10.1371/journal.pmed.1002569)
Supplement: S4 Table — (DOCX) [file pmed.1002569.s012.docx]

S4 Table. Details of patients with febrile UTI or pyelonephritis not classified as serious adverse events, intention to treat population of women with uncomplicated UTI randomized to either ibuprofen or pivmecillinam

| Diagnosis | Age | Onset* | Brief history | Urine culture** | Trial drug |
| --- | --- | --- | --- | --- | --- |
| Pyelonephritis | 23 | 3 | Returned on day 3 with flank pain, nausea and slight diarrhea, CRP 79. Treated with trimethoprim-sulphametoxazole on suspicion of pyelonephritis. The patient felt well on day 13 despite her baseline urine culture showing growth of *E. coli* resistant to trimethoprim-sulphametoxazole. | *E. coli* | Ibuprofen Took 8/9 capsules |
| Pyelonephritis | 34 | 5 | Returned after 5 days with continuous UTI symptoms. Baseline urine culture negative, new sample on day 5, no new treatment. Returned on day 6 with flank pain and fever, CRP 48. Treated with trimethoprim-sulphametoxazole. Second urine culture showed growth of an ESBL producing *E. coli.* Felt well on day 14, but still had slight dysuria. Treated with ciprofloxacin, full recovery within day 28. | *No significant growth* | Ibuprofen Completed treatment |
| Febrile UTI | 24 | 4 | Returned on day 4, felt feverish and complained of flank pain. CRP <5. Treated with trimethoprim-sulphametoxazole, still had symptoms on day 14, full recovery within 28 days. | *E. coli* | Ibuprofen Completed treatment |
| Febrile UTI | 18 | 5 | Returned on day 5 with continuous UTI symptoms and feeling feverish, CRP 11. Treated with pivmecillinam. Full recovery on day 11. | *E. coli* | Ibuprofen Completed treatment |
| Febrile UTI | 38 | 4 | Returned day 4 with continuous UTI symptoms, got a prescription for antibiotics, but did not take it. Returned again on day 6, CRP 30, received treatment with pivmecillinam. Not well by day 14, but full recovery by day 28. | *E. coli* | Ibuprofen Completed treatment |
| Febrile UTI | 21 | 4 | Returned day 4 with continuous symptoms and feeling feverish, CRP 36. Treated with pivmecillinam. Full recovery on day 9 despite baseline urine culture showing growth of *S. saprophyticus*, intrinsically resistant to pivmecillinam. | *S. saprophyticus* | Ibuprofen Completed treatment |
| Febrile UTI | 21 | 1 | Returned on day 1 with continuous UTI symptoms and slight flank pain, no fever, CRP 6. Treated with trimethoprim-sulphametoxazole. Returned on day 3 not feeling better, advised to continue given treatment. Full recovery on day 14. | *S. saprophyticus* | Ibuprofen Took 5/9 capsules |

*number of days from inclusion

**baseline urine culture
